# Machine Learning Assisted Hit Prioritization for High Throughput Screening in Drug Discovery

**DOI:** 10.1021/acscentsci.3c01517

**Published:** 2024-03-15

**Authors:** Davide Boldini, Lukas Friedrich, Daniel Kuhn, Stephan A. Sieber

**Affiliations:** † TUM School of Natural Sciences, Department of Bioscience, Center for Functional Protein Assemblies (CPA), Technical University of Munich, 85748 Garching bei München, Germany; § The Healthcare business of Merck KGaA, 64293 Darmstadt, Germany

## Abstract

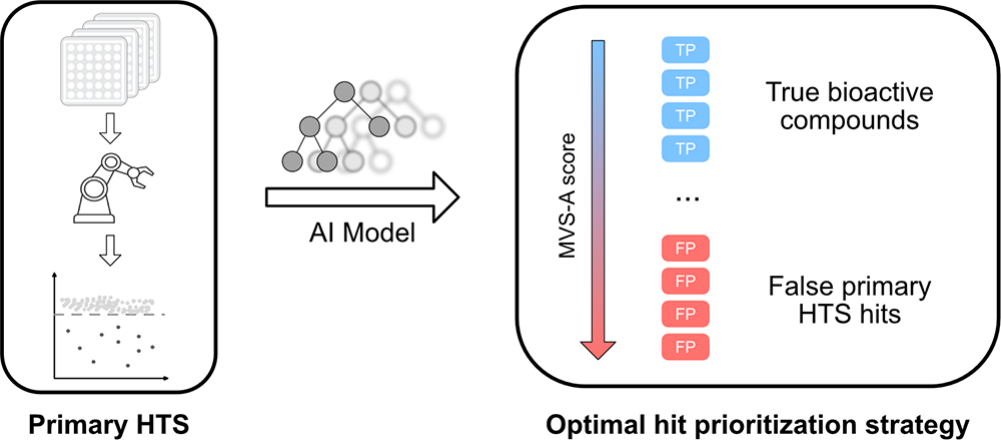

Efficient prioritization of bioactive compounds from high throughput screening campaigns is a fundamental challenge for accelerating drug development efforts. In this study, we present the first data-driven approach to simultaneously detect assay interferents and prioritize true bioactive compounds. By analyzing the learning dynamics during training of a gradient boosting model on noisy high throughput screening data using a novel formulation of sample influence, we are able to distinguish between compounds exhibiting the desired biological response and those producing assay artifacts. Therefore, our method enables false positive and true positive detection without relying on prior screens or assay interference mechanisms, making it applicable to any high throughput screening campaign. We demonstrate that our approach consistently excludes assay interferents with different mechanisms and prioritizes biologically relevant compounds more efficiently than all tested baselines, including a retrospective case study simulating its use in a real drug discovery campaign. Finally, our tool is extremely computationally efficient, requiring less than 30 s per assay on low-resource hardware. As such, our findings show that our method is an ideal addition to existing false positive detection tools and can be used to guide further pharmacological optimization after high throughput screening campaigns.

## Abstract 2

Minimum variance sampling analysis (MVS-A) is a fast machine-learning approach enabling the identification of both true bioactive compounds and false positives in high throughput screening data.

## Introduction

High throughput screening (HTS) has significantly accelerated drug discovery efforts by allowing researchers to test large chemical libraries for bioactivity in a time and cost efficient manner, thus providing a crucial starting point for synthesizing small molecules with suitable pharmacological properties.^1−4^

However, one fundamental issue with HTS is its tendency to provide false positive readouts, either because the experimental response for a given hit compound is not reproducible or because it is not correlated with the intended biological activity.^5−9^ The underlying causes for the false positive readout can be extremely heterogeneous, including colloidal aggregation,^10^ autofluorescence,^11^ interference with assay technology,^5,8^ chemical reactivity,^12^ metal impurities,^13^ and measurement uncertainty.^14^

For these reasons, choosing which active compounds to prioritize for further pharmacological development after an HTS campaign still relies on further experimental profiling,^15−17^ thus increasing the time and resources necessary to identify true hits and subsequently deliver a drug to the market.

This issue has garnered significant attention in the cheminformatics community, leading to the development of several in-silico tools for false positive detection in HTS data.^6,7,18−22^ These methods are generally based on expert rule based approaches, for example Pan-Assay Interferent (PAINS) substructure filters,^5,8^ or machine learning models trained on historical HTS data.^7,18,19^ However, there are two main limitations to the use of these tools. First, they generally make assumptions concerning the assay interference mechanism, limiting their applicability to a narrow selection of false positives.^6,18^ Furthermore, this aspect also limits their trustworthiness in identifying true positives since they can only prioritize compounds that are unlikely to be interferents according to that specific mechanism. For example, given an autofluorescence predictor for HTS interferent detection, even if it classifies a compound as nonfluorescent, that molecule might still be a false positive due to other phenomena, e.g., statistical fluctuations or colloidal aggregation. Second, these approaches depend on the chemical, biological, and technological space evaluated to generate them.^5,7^ As such, their performance might be unreliable when evaluating compounds outside of the applicability domain of the model or when applied to HTS campaigns targeting unseen protein families, relying on new assay technologies and so forth.^7^

To speed up HTS hit triaging, we propose herein minimal variance sampling analysis (MVS-A), the first machine learning approach to simultaneously identify false positive compounds and prioritize true biologically active molecules in HTS data. Our approach is inspired by recent findings in gradient-based data valuation,^23−25^ which showcase how tracing sample gradients during the training process can highlight mislabeled data in computer vision and natural language processing applications.^23,26^ To make gradient-based data valuation more applicable out-of-the-box and reduce computational complexity, MVS-A is based on a novel formulation of sample influence for gradient boosting, thus enabling processing of large HTS data sets (e.g., above 300.000 compounds) in mere seconds. Because of this, MVS-A operates in an orthogonal fashion to prior false positive detection tools for HTS data: instead of requiring a preexisting library of assay interferents, it only requires training on the HTS itself, avoiding out-of-domain (OOD) applicability issues altogether. Additionally, since it does not make any assumptions about the interference mechanism, it can be used to successfully prioritize true positives.

To evaluate our approach, we curated a selection of 17 publicly available HTS data sets and 3 industrial ones with different sizes, class imbalance, biological targets, assay technology, and false positive rates. Our results show that MVS-A can outperform a variety of rule-based and data-driven baselines both at true positive and false positive identification.

## Results and Discussion

### Using MVS-A to Prioritize Hits from HTS Campaigns

In recent years, analyzing sample gradient dynamics during supervised neural network training has attracted significant interest for modeling noisy data sets.^23−28^ These methods enable quantification of the influence of each sample on the neural network weights once the model has been trained. When training on noisy data, such as HTS campaigns, it has been shown that sample influence correlates with the likelihood of being mislabeled, thus enabling the identification of both trustworthy and problematic samples. However, neural network based approaches are computationally expensive and sensitive to hyperparameters, especially for large, imbalanced molecular data sets such as HTS data,^29,30^ making their use for nonexperts particularly challenging.

To tackle these limitations, we have developed minimum variance sampling analysis (MVS-A) to estimate sample influence in gradient boosting machines (GBM). GBM is a machine learning algorithm that fits an ensemble of decision trees in a sequence, each compensating for the mistakes of the previous tree. The advantages of using GBM instead of neural networks for computing sample influence are faster computation of importance scores, robust out-of-the-box performance, and classification performance on imbalanced HTS data, thus providing a good inductive bias for detecting false positive compounds.^31,32^ In practice, the way MVS-A works is by quantifying how “unusual” a certain active compound is according to the GBM model when comparing it to the boundary it has learned to separate active and inactive molecules. If a compound is labeled as active in the training set, but the pattern learned by the GBM model contradicts that, it will have a high MVS-A score. Vice versa, if a bioactive molecule is easily identified as such by the classifier, it will have a low MVS-A score. These scores can be used accordingly to prioritize compounds for further testing, or a threshold can be set to label true positives and false positives depending on the hit validation budget. In this study, we consider for all data sets the bottom 10% of the hits as true positives and the top 10% as false positives, as done in another ranking evaluation study.^33^

**Figure 1:**
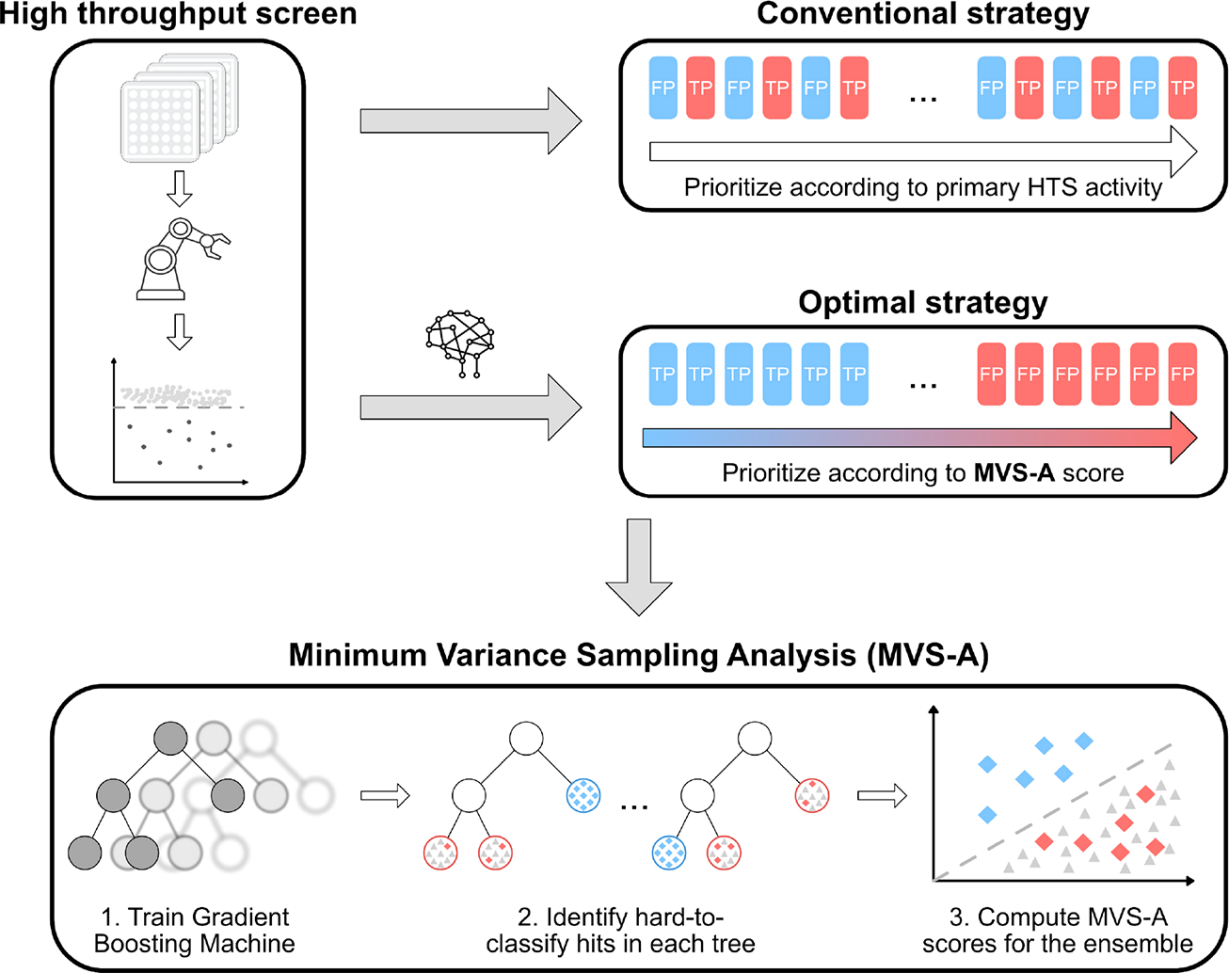
Illustration of our proposed approach. After an HTS campaign is carried out, the most active compounds in the primary screen are usually prioritized for further testing. However, this strategy often does not distinguish well between true positives (TP) and false positives (FP), leading to high false positive rates in the confirmatory screens. In our approach, we first fit a Gradient Boosting Machine classifier on the primary HTS screen data and compute MVS-A scores for each active compound. Problematic compounds according to the classifier will have high MVS-A scores and are likely false positives and vice versa for true hits. Selecting compounds according to their MVS-A score leads to reduced false positive rates in subsequent confirmatory screens and enables the identification of false positives in the primary HTS screen.

As such, our proposed approach for ranking HTS hits goes as follows (Figure 1):

(1) We train a GBM classifier on the HTS data set of interest to distinguish hits from inactive compounds.
(2) We compute sample influence estimates for all hits via MVS-A.
(3) We sort all HTS hits according to their MVS-A score. False positives are likely to have high MVS-A scores and vice versa for true positives.

Thanks to its computational efficiency, this pipeline takes only a few seconds on low-end hardware, even for large HTS data sets. Crucially, our approach relies exclusively on the HTS data set of interest. As such, it does not rely on historical information on which compounds tend to be false positives for that assay technology (like, e.g., PAINS), nor on assumptions of which biophysical process is causing the interference (e.g., aggregation or autofluorescence predictors). This means that our method is inherently applicable to any assay technology and any region of the chemical space while being able to detect any type of interferent.

Finally, we provide a more in-depth discussion of the theory behind MVS-A in chapter 1 of the Supporting Information.

### Constructing a Benchmark for HTS Hit Prioritization

To evaluate our proposed approach, we curated a selection of 17 data sets from publicly available HTS data,^34,35^ for a total of 471370 unique compounds measured against 10 different protein families, using a variety of readout measurements and activity thresholds (Table 1, Table S1, Table S2, Table S3, and Table S4). We focused on HTS data sets where more than 200 hits were investigated both in primary and confirmatory screens, excluding campaigns where the false positive rate was above 95% or below 5%. Where possible, we prioritized the selection of assays targeting different protein families and confirmatory screen protocols.

**Table 1:** Summary Information for the Datasets Employed in This Study^a^

name	source	number of compounds	false positive %	number of hits
transporter	ref (33)	306252	29%	2625
transcription	ref (33)	344724	47%	2336
transcription_2	ref (33)	301125	76%	2325
GPCR_2	ref (33)	196068	79%	1980
GPCR_3	ref (33)	63643	56%	2176
ion_channel	ref (33)	305411	15%	2580
ion_channel_2	ref (33)	104663	21%	4227
ion_channel_3	ref (33)	305401	32%	1642
kinase	ref (34)	321563	21%	234
GPCR	ref (34)	325747	51%	5742
serine	ref (34)	214071	91%	1262
transcription_3	ref (34)	363477	81%	1790
ubiquitin	ref (34)	330197	70%	1533
splicing	ref (34)	293183	11%	2189
channel_atp	ref (34)	343522	48%	1229
cysteine_protease	ref (34)	344098	48%	1842
zinc_finger	ref (34)	301590	48%	1132

a The number of hits defines the number of active compounds in the primary screen. The false positive percentage identifies the fraction of active compounds in the primary screen that were found to be inactive in the confirmatory screen.

As a result of this selection process, the false positive rates in our benchmark range from 11% to 91%, and the screened libraries evaluate different regions of the chemical space (Figure S1), thus covering a broad spectrum of HTS campaigns.

Each data set is generated from a primary screen, relying on single-dose measurements, and a confirmatory screen, which either adds replicates or assesses the dose–response activity against the same biological target. To define which molecules are considered bioactive in a given primary or confirmatory screen, we employed the original activity thresholds defined by the authors of the screening campaign. This ensures that our analysis accurately reflects real drug discovery campaigns as close as possible, where bioactivity criteria vary on a case-by-case basis, depending on the biological target and the purpose of the drug.

We define a compound as false positive if it was reported to be active in the primary screen but was found to be inactive or inconclusive in the confirmatory screen. Depending on the protocol employed for the confirmatory screen, different false positive types can be identified. When adding replicates, only errors associated with readout fluctuations or systematic errors (e.g., dust in the well plate) can be identified, while dose–response measurements enable detection of autofluorescence, colloidal aggregation, assay technology interference, and so forth.

### Defining a Protocol to Assess HTS Hit Prioritization Strategies

For a given HTS data set, we run the MVS-A pipeline exclusively on the primary screening data. Then, we measure how effective our approach is at separating true actives and false positives by comparing its compound ranking to the confirmatory screening data. To evaluate the sorting performance, we measure top-K Precision, Enrichment Factor, and Boltzmann-Enhanced Discrimination of Receiver Operating Characteristic (BEDROC).^33^ Since Precision is sensitive to the amount of noise in the data set, we scale it with respect to the false positive and true positive rate for each data set, making this metric more consistent across data sets. Therefore, a relative top-K precision score of 0.0 indicates that a given ranking is equal to random sorting, while values above 0.0 denote percent improvements over assay noise. We further discuss our metric selection in chapter 4 of the Supporting Information.

To contextualize the performance of MVS-A, we provide the following baselines:

- Detecting false positives according to REOS and GSK structural filters, two well-established rule-based approaches to detect false positives in HTS data.^36,37^ We rank compounds in terms of the total number of flags according to both criteria.
- Prioritizing compounds for further screening according to activity in the primary HTS assay, the defacto approach for ranking hits both in academia and in the industry.^15^ The underlying assumption here is that if a compound is very active in the primary screen, it is likely to have similar bioactivity in the confirmatory screen as well.
- Ranking according to CatBoost object importance,^38^ another sample influence approach based on GBM relying on a different algorithm to compute importance scores. We discuss this method further in the Supporting Information.
- Ranking according to Isolation Forest, a well-established anomaly detection algorithm based on decision tree ensembles.^39^ We use the default parameters from the Scikit-Learn package.^40^
- Ranking according to the reconstruction error of a Variational Autoencoder (VAE), a popular deep learning approach for anomaly detection.^41−43^ We implement a SMILES-based VAE using the architecture described by Gómez-Bombarelli et al.^44^

Additionally, to compare our approach with publicly available HTS interference predictors, we add the following baselines for false positive identification:

- Hit Dexter 3 (HD) for frequent hitter prediction.^6,18,45^
- SCAM Detective for colloidal aggregator identification.^46^
- An in-house autofluorescence predictor based on the models used by InterPred.^22^ We discuss how we reproduced their featurization and optimization procedure in the Supporting Information.

### MVS-A Achieves Best Performance in HTS False Positive Detection

**Figure 2:**
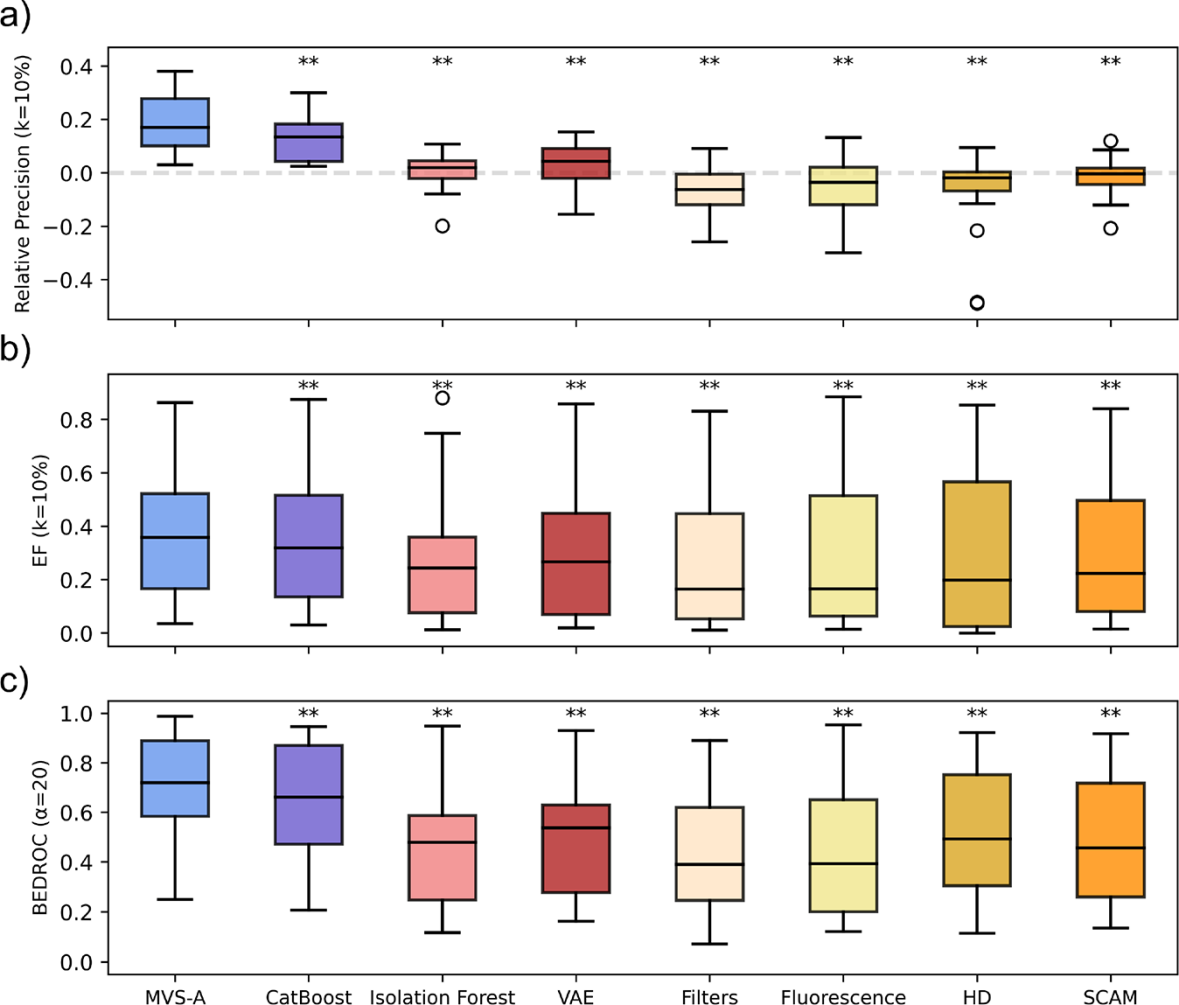
False positive detection performance across all data sets. Asterisks denote significance according to one-tailed Wilcoxon Signed Rank tests with Bonferroni correct (one asterisk corresponds to α = 0.05, two asterisks to α = 0.01). *P*-values are reported in Table S5. (a) Distribution of relative precision scores across all data sets. The dotted gray line denotes random performance. (b) Distribution of enrichment factor scores across all data sets. (c) Distribution of BEDROC scores across all data sets.

In terms of false positive detection, MVS-A matches or outperforms, on average, all alternative methods across all metrics (Figure 2). The performance of our approach is mostly consistent across different metrics, meaning that MVS-A provides the best performance both when considering the top 10% predictions, as indicated by relative precision and enrichment factor, and when evaluating the entire ranking, as measured by BEDROC. Crucially, MVS-A outperforms all baselines across all metrics and data sets on 12 out of 17 data sets, while achieving second best performance on the remaining 5, making it an ideal option for out-of-the-box scenarios.

CatBoost object importance is the most competitive alternative; however, MVS-A still outperforms it on 16 out of 17 data sets across all metrics. Compared to this baseline, MVS-A provides an improvement of 29%, 6%, and 10% for relative precision, enrichment factor, and BEDROC respectively. Considering the differences in sample importance formulation between these methods, this result supports our method’s assumption that focusing on the splitting decisions provides a better inductive bias for discovering mislabeled data.

Both anomaly detection methods, namely, Isolation Forest and VAE, struggle on this benchmark. Specifically, MVS-A outperforms them across all metrics on 16/17 and 15/17 data sets, respectively. Concerning VAE, this is likely due to the fact that these algorithms require large data sets (e.g., 10^6^ compounds) to be trained properly,^44^ while the number of hits per HTS is much lower. Regarding Isolation Forest, its performance is likely affected by the high dimensionality of the input molecular representations, rendering the use of random splits less effective.^39^ In contrast, data valuation approaches like MVS-A object importance select the subset of informative features by first fitting a supervised classifier to distinguish active and inactive compounds, thus mitigating the issue of high dimensionality.

In comparison to GSK and REOS structural filters, MVS-A outperforms them on 16/17 data sets across all metrics. However, these alerts do not only detect false positives, but also focus on chemical moieties associated with target promiscuity or other undesirable pharmacological properties.^36,47,48^ This mismatch then could explain the poor performance observed in identifying false positives in this benchmark.

Finally, Hit Dexter, SCAM Detective, and the autofluorescence predictor show subpar false positive detection performance when compared to MVS-A, with our approach outperforming them across all metrics on 16/17, 17/17, and 15/17 data sets, respectively. This is likely because these approaches, unlike MVS-A, focus on specific interference mechanisms, while our benchmark makes no assumptions about the false positive origin. Furthermore, the performance of these baselines is likely degraded by applicability domain issues, while MVS-A is tailored to each specific screening campaign.

### MVS-A Provides the Most Efficient HTS True Hit Prioritization Strategy

**Figure 3:**
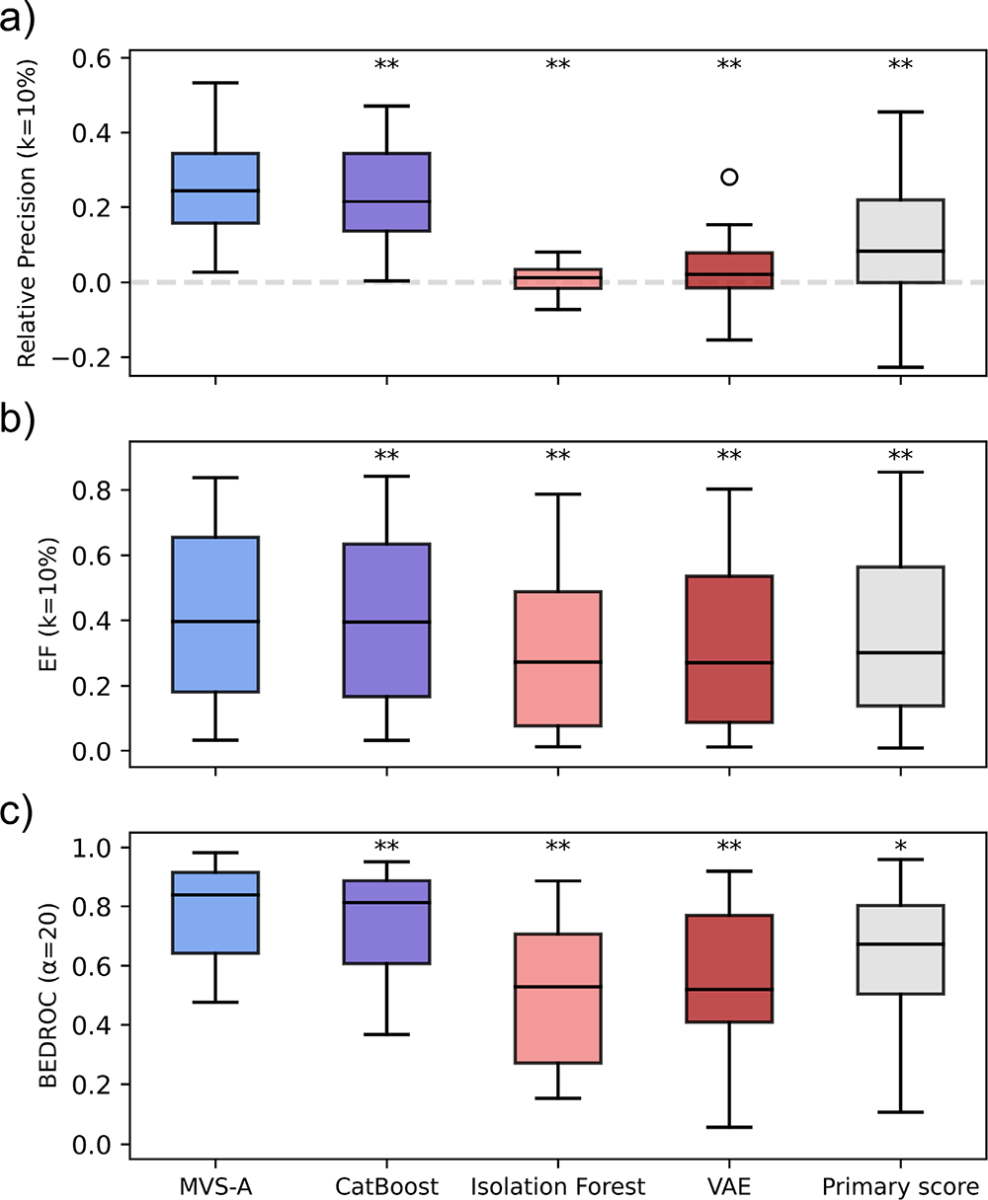
True positive detection performance across all data sets. Asterisks denote significance according to one-tailed Wilcoxon Signed Rank tests with Bonferroni correct (one asterisk corresponds to α = 0.05, two asterisks to α = 0.01). *P*-values are reported in Table S6. (a) Distribution of relative precision scores across all data sets. The dotted gray line denotes random performance. (b) Distribution of enrichment factor scores across all data sets. (c) Distribution of BEDROC scores across all data sets.

In line with the false positive retrieval benchmark, MVS-A on average matches or outperforms all other approaches across all metrics in terms of true hit detection (Figure 3) Specifically, it achieves the best performance in 13 data sets out of 17 across all metrics, and it ranks second best in the remaining 4 data sets, further highlighting its potential as an optimal out-of-the-box solution.

On average, the most competitive baseline is again CatBoost object importance; however, MVS-A still outperforms it on 16/17 data sets. This further highlights that MVS-A is more effective at assessing sample influence than the previous state-of-the-art GBM algorithms since it detects high fidelity data more efficiently.

As for the false positive detection benchmark, anomaly detection methods provide subpar performance for true positive identification, with VAE showing slightly better performance than Isolation Forest. This is likely a consequence of the low data available for training the VAE and the high dimensionality of the input in the case of the Isolation Forest.

Finally, in comparison to the primary readout ranking, MVS-A outperforms it on 15 data sets, with average improvements of 50%, 13%, and 14% in terms of relative precision, enrichment factor, and BEDROC. This is especially impressive considering that ranking compounds according to their primary HTS readout is the industry standard for hit triaging in HTS campaigns. The relatively low performance of this method could be due to the fact that assay interferents can be outliers in terms of primary readout, for example, by exhibiting very strong autofluorescence, causing them to be at the top of the primary readout ranking. As such, this benchmark shows that our data-driven approach is more efficient at finding true actives than the currently used criteria for HTS hit triaging

### MVS-A Identifies Structurally Diverse Interferents

While being able to correctly prioritize true positives and exclude false positives is a fundamental requirement for an HTS hit triaging strategy, retrieving a diverse set of compounds is also crucial. To assess this, we investigated the ability of our approach to identify heterogeneous true actives and assay interferents by measuring the fraction of unique Murcko scaffolds among the hits for both categories in each data set.

**Figure 4:**
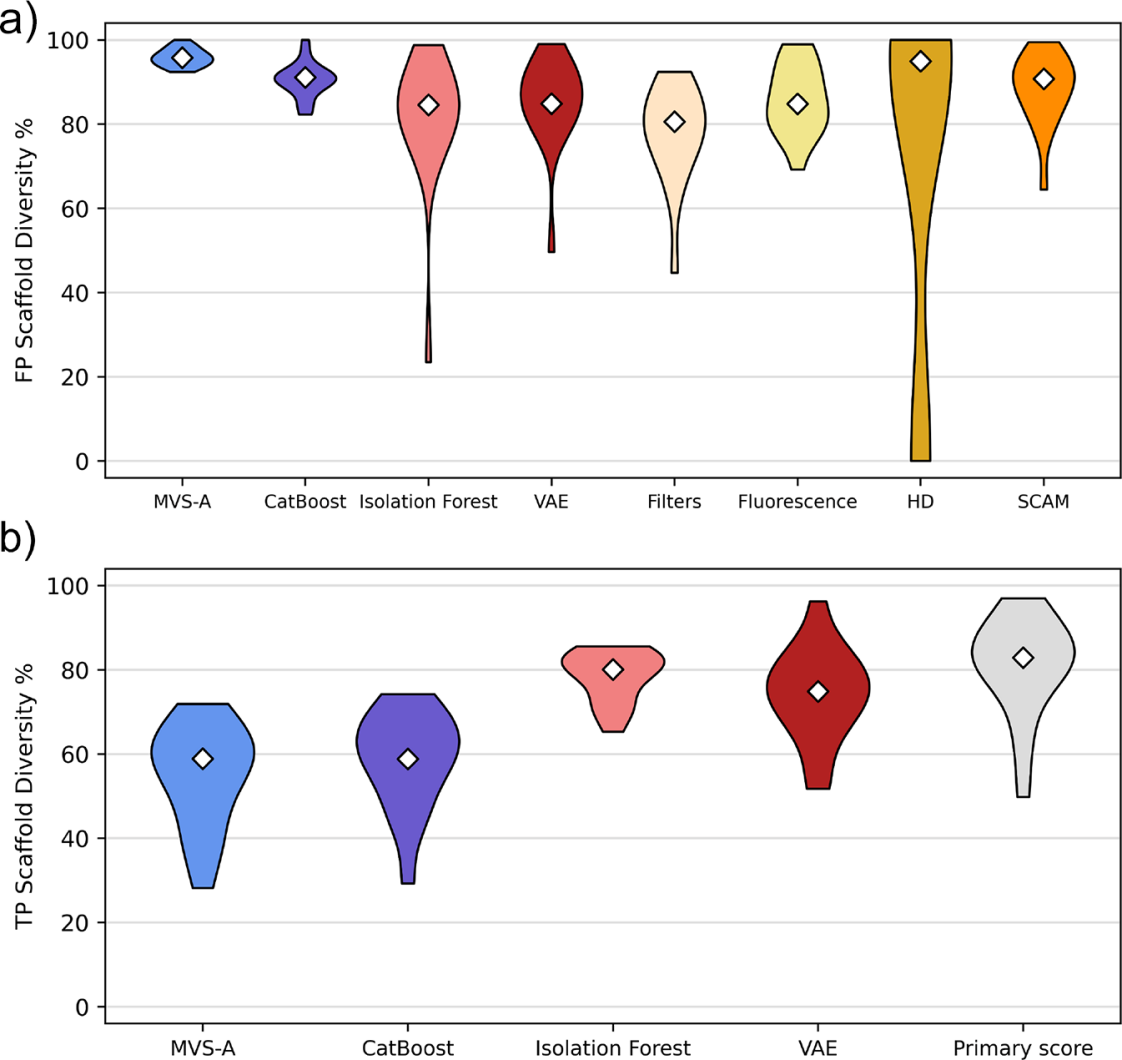
Structural diversity distribution analysis. White diamonds indicate the median of the distribution. (a) Distribution of the scaffold diversity scores across all data sets for false positive detection. (b) Distribution of the scaffold diversity scores across all data sets for true positive detection.

In terms of false positive variety, MVS-A selects the most diverse selection of interferents, peaking at around 95% scaffold diversity, closely followed by Hit Dexter and CatBoost object importance (Figure 4a). In general, data valuation algorithms such as MVS-A naturally tend to identify more varied interferents since they do not rely on the presence of specific molecular motifs in the false positives but rather highlight any active that deviates from the pattern they learned while training on the primary screening data. This more flexible definition of what constitutes a false positive then leads to the identification of more structurally different interferents, outperforming even anomaly detection algorithms. On the contrary, structural filters and assumption-based predictors are inherently biased toward specific chemical scaffolds, thus flagging more homogeneous compounds. One exception to this seems to be frequent hitters, which likely encompass several different interference mechanisms in their definition and, as such, have more diverse chemical structures.

This trend is inverted for true positive discovery, where both data valuation approaches tend to yield less diverse selections of true hits, centered around 60% scaffold diversity (Figure 4b). In this case, the true positives identified by MVS-A and CatBoost are the ones that fit well the learned class boundary between actives and inactives in the primary data. The boundary in this case tends to include only a limited region of the chemical space, leading to more structurally similar true actives. In contrast, primary readout ranking has no chemical bias in its selection criteria, thus retrieving the most diverse true positives. Finally, the scaffold diversity rate distribution across all data sets for the anomaly detection baselines is comparable with the one observed to randomly picking hits from each HTS (Table S4). This is because the true positives identified by these methods correspond to distribution inliers, thus approximating the distribution of chemical motifs present in the training data.

### MVS-A Identifies False Positives Belonging to Different Interferent Classes

By design, MVS-A makes no assumption concerning the interference mechanism of the false positive compounds in the primary screen; therefore, it should cover all types of interferents. To test this assumption, we measure across all data sets the fraction of compounds predicted to be false positives by our method that were also identified by the other assumption-based predictors (Figure S2).

MVS-A shows the highest overlap across all data sets with the autofluorescence predictor, with a median of 66%. This however is likely due to the nonselectivity of the autofluorescence predictor, which tends to flag the majority of compounds as fluorescent across all data sets (Table S7). These overconfident predictions could be due to applicability domain issues given that the training set used for this model originated from assays related to toxicological screening. Compared to the remaining in-silico predictors, MVS-A shows a median overlap of 51% with the colloidal aggregators identified by SCAM Detective, 33% with the structural filters from GSK and REOS and 8% with the frequent hitters detected by Hit Dexter.

Taken together, these results confirm the hypothesis that MVS-A can identify different classes of false positives while showing complementary performance with tools covering also compound promiscuity, such as frequent hitter predictors and general nuisance compound structural alerts.

### Case Study I: Choline Transporter Inhibitor Screen from Vanderbilt University

To assess how well MVS-A would perform in a real drug discovery campaign, we investigated whether the true actives identified by our method are biased toward chemical moieties that would make them unsuitable for further pharmacological optimization. To do so, we re-evaluated the data set with the codename “transporter” from the publicly available HTS assays evaluated in this work. This assay was conducted in order to identify novel inhibitors for the presynaptic choline transporter (CHT), a potential therapeutic target for Alzheimer’s disease and schizophrenia.^49^ We chose this data set from our collection as a case study because the hits from its primary HTS screen were extensively validated by additional counterscreens and confirmatory assays (PubChem AID 488997). The goal of these experimental validation efforts was to identify potent selective CHT inhibitors eliciting the desired phenotypic response from primary HTS hits.

**Figure 5:**
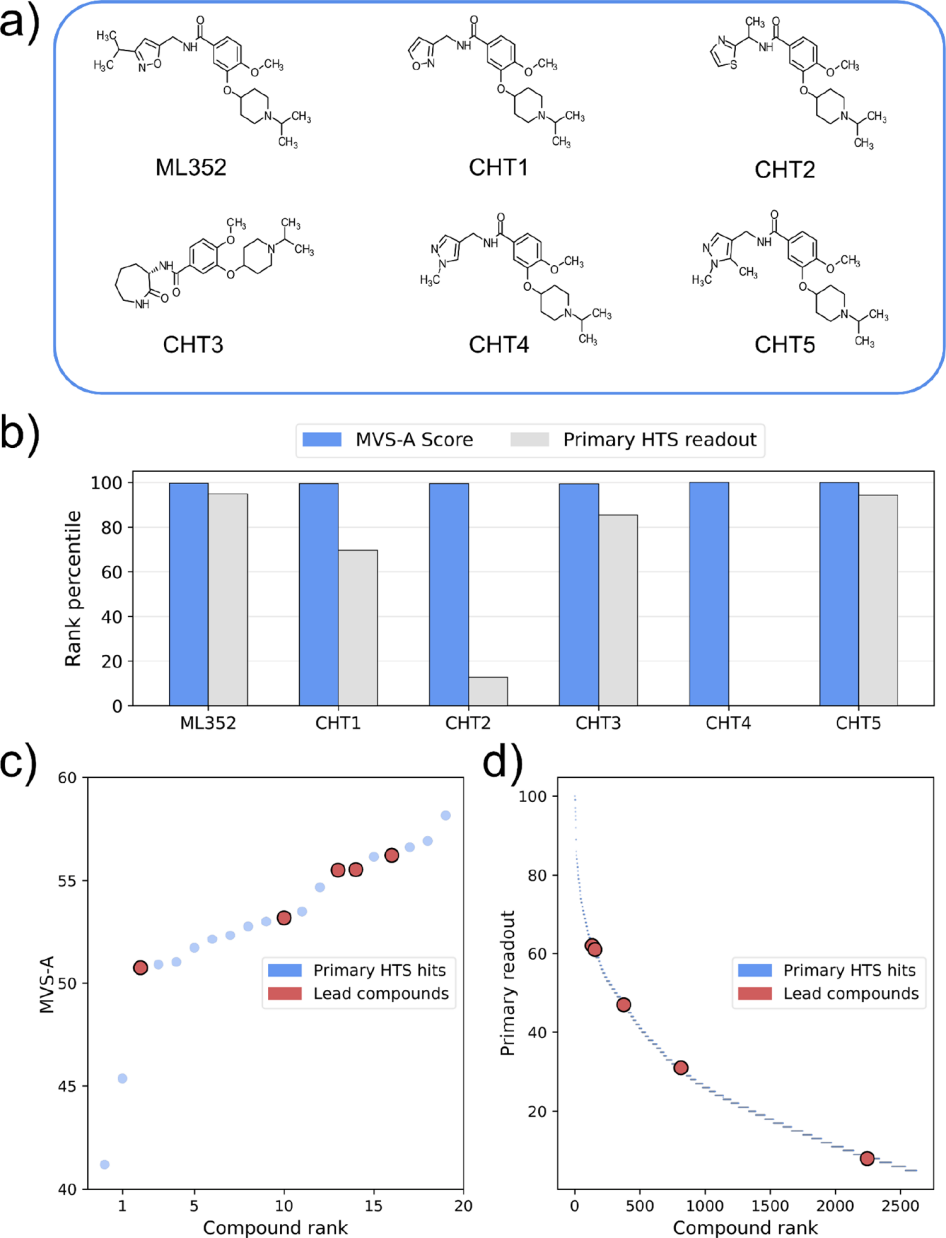
(a) Structures of the most relevant true positive compounds from the choline transporter inhibitor screening campaign. (b) Rank percentiles for the lead compounds according to MVS-A and the experimental readout from the primary HTS. (c) Compound ranking for the primary hits according to MVS-A. (d) Compound ranking according to the experimental readout for the primary hits.

After 11 rounds of screening, only six compounds that were present in the primary HTS made it to the end of the pipeline, one of which, CHT4, was a false negative (Figure 5a). Crucially, all five true positives were immediately flagged as more promising than most other hits from the HTS campaign by MVS-A (Figure 5b), ranking within the top 20 primary HTS according to our method. Notably, among these five compounds there was also ML352, the best inhibitor from the screening campaign, which also showed suitable ADME properties.^49^ In contrast, ranking by experimental readout from the primary screen is far less efficient, with ML352, CHT5, and CHT3 ranking between 150th and 500th, CHT1 around 750th, and CHT2 around 2300th (Figure 5b,d).

We then used MVS-A to rank primary inactive compounds in terms of false negative likelihood by sorting inactive compounds in terms of importance to the underlying GBM classifier according to our method. Crucially, our approach correctly identified CHT4 as the most likely false negative compound out of all primary inactives (Figure 5b). This finding is especially relevant, since mining dark chemical matter in HTS data is a promising but largely unexplored starting point for drug discovery,^50^ given the lack of in-silico approaches to determine which samples to reinvestigate.

To summarize, in this case study MVS-A was able to identify the 6 most biologically relevant compounds just by observing the primary HTS data, including a false negative, while prioritizing molecules according to their experimental readout in the primary screen was a less efficient selection strategy. Additionally, this finding shows that MVS-A is not biased toward undesirable chemical moieties in terms of further pharmacological development.

### Case Study II: Industrial HTS Campaigns from Merck KGaA

To further evaluate the applicability of MVS-A on real scenarios, we investigated three currently ongoing HTS campaigns from Merck KGaA, aimed at different biological targets (Table S8). Each of these data sets is larger than the largest publicly available data set we included in our study so far, thus providing a realistic benchmark for how our method would fare in industrial applications. Due to computational limitations, we could only test MVS-A, CatBoost and primary readout ranking on these data sets.

In terms of false positive detection, on average, MVS-A outperforms all baselines across all metrics (Table S9). Regarding true positive detection, on average, CatBoost and MVS-A achieve similar performance, while primary readout ranking outperforms all alternatives in terms of precision and BEDROC (Table S10). In general, primary readout ranking performs much better as a baseline in these data sets, likely due to less assay noise compared to publicly available data, making the initial HTS screen more predictive of a compound’s performance in further validation screens.

### Limitations and Practical Guidelines for the Use of MVS-A

While MVS-A achieved excellent performance in terms of false positive and true positive detection, it still requires careful deployment for real use cases.

First, the performance of MVS-A can fluctuate from data set to data set, and it can be difficult to forecast how effective it will be for a given HTS data set. While we investigated the relationship between its performance and the HTS of interest, such as the protein target family, structural diversity of the data set (Figure S3), and the cross-validation performance of the GBM classifier (Figure S4), we could not detect meaningful correlation between these factors. As such, although MVS-A never performs worse than random picking in our benchmarks, the bias toward specific scaffolds in terms of true positive prioritization can be problematic if it is not associated with an improved true hit rate. This issue can be tackled, however, by hybrid hit selection strategies aimed at selecting diverse chemical scaffolds according to the MVS-A true hit likelihood.

Another factor that can influence the performance is the choice of molecular representation for the analysis. However, we observed only a 3% performance variation when using different molecular fingerprints and molecular descriptors (Figure S5), consistently with the results observed for molecular property prediction tasks.

In terms of computational cost, unlike other false positive predictors, MVS-A requires to be retrained for each new HTS data set. However, our testing shows that the algorithm is extremely efficient and lightweight, taking less than 5 s per data set on a server with an AMD Ryzen Threadripper 3970X 32-Core Processor and less than 30 s on a laptop with an AMD Ryzen 5 3600 6-Core Processor (Figure S6).

Finally, while MVS-A accurately distinguishes between interferents and true positives, it does not account for other relevant factors for hit prioritization such as promiscuity. As such, the ideal application of our approach is not as a stand-alone tool, but in conjunction with other in-silico tools, e.g., structural alerts or frequent hitter predictors, to get a global view of the pharmacological potential of each primary HTS hit. To highlight this, we revisited the top 20 ranked compounds from the CHT inhibitor screening campaign according to MVS-A, as discussed in Case Study I, focusing on the 15 compounds our approach incorrectly selected as the true hit. Six of those could be removed according to REOS and GSK filters, one according to Hit Dexter and one according to InterPred, while SCAM Detective flagged most compounds as potential colloidal aggregators (Table S10). As such, the synergistic combination of these approaches could have brought the true positive rate from 25% when using MVS-A on its own to 38%.

## Conclusions

High throughput screening holds a key role in current drug discovery research, but its impact is limited by the presence of many false positive compounds, making further pharmacological development of bioactive compounds slower and more expensive. In this study, we introduced minimal variance sampling analysis, a novel approach inspired by data valuation methods to simultaneously prioritize true positive compounds and detect assay interferents in HTS data.

To test our proposed method, we have constructed a new benchmark consisting of 17 primary-confirmatory HTS data set pairs, encompassing a variety of biological targets, assay technologies, number of compounds, and false positive rates.

MVS-A consistently matches or outperforms the other baselines in terms of both false positive and true positive detection. Crucially, it provides average improvements up to 50%, 13%, and 14% in terms of relative precision, enrichment factor, and BEDROC against primary readout sorting, a popular heuristic used in the pharmaceutical industry for HTS hit prioritization. Concerning false positive discovery, our method can identify a wide range of structurally diverse interferents with low overlap with the predictions of prior in-silico tools focusing on compound promiscuity, making our method an excellent addition to HTS false positive detection pipelines.

Regarding hit prioritization, MVS-A was able to identify the most biologically relevant hits from a primary HTS campaign in a retrospective case study on publicly available data. Interestingly, one of the hits correctly detected by MVS-A was a false positive, highlighting the potential of our approach to detect promising compounds from dark chemical matter.

On the three data sets provided by Merck KGaA, MVS-A performs competitively in terms of false positive detection and false positive retrieval, indicating that our approach is also reliable in the chemical space typically explored in industrial screening campaigns.

Finally, our method is extremely computationally efficient, allowing processing of HTS data on a laptop in under 30 s with minimal RAM usage. In light of these results, we are confident MVS-A will help accelerate HTS hit triaging and will stimulate further research into data valuation approaches for handling large chemical data sets. We provide this tool as an open source package at https://github.com/dahvida/AIC_Finder.

## Data Availability

All PubChem assays investigated in this study can be accessed from PubChem according to their AID, as shown in Table 1. The Python environment, data sets, performance of each method across all metrics, data sets and replicates, and code required to reproduce the results are available at the following GitHub repository: https://github.com/dahvida/AIC_Finder.

## Supporting Information Available

Technical explanation of the theoretical aspects of MVS-A, the Methods section detailing how each approach was implemented, training time measurements, chemical space diversity analysis, false positive identification performance for the alternative machine-learning approaches, overlap analysis between the predictions of MVS-A and alternative machine-learning approaches (PDF)

## References

[ref1] Blay V.; Tolani B.; Ho S. P.; Arkin M. R. High-Throughput Screening: Today’s Biochemical and Cell-Based Approaches. Drug Discovery Today 2020, 25 (10), 1807–1821. 10.1016/j.drudis.2020.07.024. 32801051

[ref2] Kim S.; Chen J.; Cheng T.; Gindulyte A.; He J.; He S.; Li Q.; Shoemaker B. A.; Thiessen P. A.; Yu B.; Zaslavsky L.; Zhang J.; Bolton E. E. PubChem in 2021: New Data Content and Improved Web Interfaces. Nucleic Acids Res. 2021, 49 (D1), D1388–D1395. 10.1093/nar/gkaa971. 33151290 PMC7778930

[ref3] Macarron R.; Banks M. N.; Bojanic D.; Burns D. J.; Cirovic D. A.; Garyantes T.; Green D. V. S.; Hertzberg R. P.; Janzen W. P.; Paslay J. W.; Schopfer U.; Sittampalam G. S. Impact of High-Throughput Screening in Biomedical Research. Nat. Rev. Drug Discovery 2011, 10 (3), 188–195. 10.1038/nrd3368. 21358738

[ref4] Schneider P.; Walters W. P.; Plowright A. T.; Sieroka N.; Listgarten J.; Goodnow R. A.; Fisher J.; Jansen J. M.; Duca J. S.; Rush T. S.; Zentgraf M.; Hill J. E.; Krutoholow E.; Kohler M.; Blaney J.; Funatsu K.; Luebkemann C.; Schneider G. Rethinking Drug Design in the Artificial Intelligence Era. Nat. Rev. Drug Discovery 2020, 19 (5), 353–364. 10.1038/s41573-019-0050-3. 31801986

[ref5] Dahlin J. L.; Nissink J. W. M.; Strasser J. M.; Francis S.; Higgins L.; Zhou H.; Zhang Z.; Walters M. A. PAINS in the Assay: Chemical Mechanisms of Assay Interference and Promiscuous Enzymatic Inhibition Observed during a Sulfhydryl-Scavenging HTS. J. Med. Chem. 2015, 58 (5), 2091–2113. 10.1021/jm5019093. 25634295 PMC4360378

[ref6] Stork C.; Chen Y.; Šícho M.; Kirchmair J. Hit Dexter 2.0: Machine-Learning Models for the Prediction of Frequent Hitters. J. Chem. Inf. Model. 2019, 59 (3), 1030–1043. 10.1021/acs.jcim.8b00677. 30624935

[ref7] David L.; Walsh J.; Sturm N.; Feierberg I.; Nissink J. W. M.; Chen H.; Bajorath J.; Engkvist O. Identification of Compounds That Interfere with High-Throughput Screening Assay Technologies. ChemMedChem. 2019, 14 (20), 1795–1802. 10.1002/cmdc.201900395. 31479198 PMC6856845

[ref8] Baell J. B.; Nissink J. W. M. Seven Year Itch: Pan-Assay Interference Compounds (PAINS) in 2017—Utility and Limitations. ACS Chem. Biol. 2018, 13 (1), 36–44. 10.1021/acschembio.7b00903. 29202222 PMC5778390

[ref9] Sink R.; Gobec S.; Pečar S.; Zega A. False Positives in the Early Stages of Drug Discovery. Curr. Med. Chem. 2010, 17 (34), 4231–4255. 10.2174/092986710793348545. 20939815

[ref10] Auld D. S.; Inglese J.; Dahlin J. L. Assay Interference by Aggregation. In Assay Guidance Manual; Markossian S., Grossman A., Brimacombe K., Arkin M., Auld D., Austin C., Baell J., Chung T. D. Y., Coussens N. P., Dahlin J. L., Devanarayan V., Foley T. L., Glicksman M., Gorshkov K., Haas J. V., Hall M. D., Hoare S., Inglese J., Iversen P. W., Kales S. C., Lal-Nag M., Li Z., McGee J., McManus O., Riss T., Saradjian P., Sittampalam G. S., Tarselli M., Trask O. J., Wang Y., Weidner J. R., Wildey M. J., Wilson K., Xia M., Xu X., Eds.; Eli Lilly & Company and the National Center for Advancing Translational Sciences: Bethesda (MD), 2004. 28749639

[ref11] Hall M. D.; Simeonov A.; Davis M. I. Avoiding Fluorescence Assay Interference—The Case for Diaphorase. Assay Drug Dev. Technol. 2016, 14 (3), 175–179. 10.1089/adt.2016.707. 27078679 PMC4840916

[ref12] Huth J. R.; Mendoza R.; Olejniczak E. T.; Johnson R. W.; Cothron D. A.; Liu Y.; Lerner C. G.; Chen J.; Hajduk P. J. ALARM NMR: A Rapid and Robust Experimental Method To Detect Reactive False Positives in Biochemical Screens. J. Am. Chem. Soc. 2005, 127 (1), 217–224. 10.1021/ja0455547. 15631471

[ref13] Hermann J. C.; Chen Y.; Wartchow C.; Menke J.; Gao L.; Gleason S. K.; Haynes N.-E.; Scott N.; Petersen A.; Gabriel S.; Vu B.; George K. M.; Narayanan A.; Li S. H.; Qian H.; Beatini N.; Niu L.; Gan Q.-F. Metal Impurities Cause False Positives in High-Throughput Screening Campaigns. ACS Med. Chem. Lett. 2013, 4 (2), 197–200. 10.1021/ml3003296. 24900642 PMC4027514

[ref14] Dragiev P.; Nadon R.; Makarenkov V. Systematic Error Detection in Experimental High-Throughput Screening. BMC Bioinformatics 2011, 12 (1), 25 10.1186/1471-2105-12-25. 21247425 PMC3034671

[ref15] Vincent F.; Loria P. M.; Weston A. D.; Steppan C. M.; Doyonnas R.; Wang Y.-M.; Rockwell K. L.; Peakman M.-C. Hit Triage and Validation in Phenotypic Screening: Considerations and Strategies. Cell Chem. Biol. 2020, 27 (11), 1332–1346. 10.1016/j.chembiol.2020.08.009. 32888500

[ref16] Hughes J.; Rees S.; Kalindjian S.; Philpott K. Principles of Early Drug Discovery. Br. J. Pharmacol. 2011, 162 (6), 1239–1249. 10.1111/j.1476-5381.2010.01127.x. 21091654 PMC3058157

[ref17] Hevener K. E.; Pesavento R.; Ren J.; Lee H.; Ratia K.; Johnson M. E. Chapter Twelve - Hit-to-Lead: Hit Validation and Assessment. In Methods in Enzymology; Lesburg C. A., Ed.; Modern Approaches in Drug Discovery; Academic Press, 2018; Vol. 610, pp 265–309. 10.1016/bs.mie.2018.09.022. 30390802

[ref18] Stork C.; Wagner J.; Friedrich N.-O.; de Bruyn Kops C.; Šícho M.; Kirchmair J. Hit Dexter: A Machine-Learning Model for the Prediction of Frequent Hitters. ChemMedChem. 2018, 13 (6), 564–571. 10.1002/cmdc.201700673. 29285887

[ref19] Yang Z.-Y.; Yang Z.-J.; Dong J.; Wang L.-L.; Zhang L.-X.; Ding J.-J.; Ding X.-Q.; Lu A.-P.; Hou T.-J.; Cao D.-S. Structural Analysis and Identification of Colloidal Aggregators in Drug Discovery. J. Chem. Inf. Model. 2019, 59 (9), 3714–3726. 10.1021/acs.jcim.9b00541. 31430151

[ref20] Irwin J. J.; Duan D.; Torosyan H.; Doak A. K.; Ziebart K. T.; Sterling T.; Tumanian G.; Shoichet B. K. An Aggregation Advisor for Ligand Discovery. J. Med. Chem. 2015, 58 (17), 7076–7087. 10.1021/acs.jmedchem.5b01105. 26295373 PMC4646424

[ref21] Lee K.; Yang A.; Lin Y.-C.; Reker D.; Bernardes G. J. L.; Rodrigues T. Combating Small-Molecule Aggregation with Machine Learning. Cell Rep. Phys. Sci. 2021, 2 (9), 100573 10.1016/j.xcrp.2021.100573.

[ref22] Borrel A.; Mansouri K.; Nolte S.; Saddler T.; Conway M.; Schmitt C.; Kleinstreuer N. C. InterPred: A Webtool to Predict Chemical Autofluorescence and Luminescence Interference. Nucleic Acids Res. 2020, 48 (W1), W586–W590. 10.1093/nar/gkaa378. 32421835 PMC7319558

[ref23] Pruthi G.; Liu F.; Sundararajan M.; Kale S. Estimating Training Data Influence by Tracing Gradient Descent. arXiv, November 14, 2020. 10.48550/arXiv.2002.08484 (accessed 2022-08-31).

[ref24] Feng Y.; Tu Y. Phases of Learning Dynamics in Artificial Neural Networks in the Absence or Presence of Mislabeled Data. Mach. Learn. Sci. Technol. 2021, 2 (4), 043001 10.1088/2632-2153/abf5b9.

[ref25] Pleiss G.; Zhang T.; Weinberger K. Q.; Elenberg E. Identifying Mislabeled Data Using the Area Under the Margin Ranking arXiv, 2020. 10.48550/arXiv.2001.10528.

[ref26] Akyurek E.; Bolukbasi T.; Liu F.; Xiong B.; Tenney I.; Andreas J.; Guu K. Towards Tracing Knowledge in Language Models Back to the Training Data. In Findings of the Association for Computational Linguistics: EMNLP 2022; Association for Computational Linguistics: Abu Dhabi, United Arab Emirates, 2022; pp 2429–2446.

[ref27] Lu Y.; Bo Y.; He W. Noise Attention Learning: Enhancing Noise Robustness by Gradient Scaling. Adv. Neural Inf. Process. Syst. 2022, 35, 23164–23177.

[ref28] Toniato A.; Schwaller P.; Cardinale A.; Geluykens J.; Laino T. Unassisted Noise Reduction of Chemical Reaction Datasets. Nat. Mach. Intell. 2021, 3 (6), 485–494. 10.1038/s42256-021-00319-w.

[ref29] Keshavarzi Arshadi A.; Salem M.; Firouzbakht A.; Yuan J. S. MolData, a Molecular Benchmark for Disease and Target Based Machine Learning. J. Cheminformatics 2022, 14 (1), 10 10.1186/s13321-022-00590-y. PMC8899453 35255958

[ref30] Korkmaz S. Deep Learning-Based Imbalanced Data Classification for Drug Discovery. J. Chem. Inf. Model. 2020, 60 (9), 4180–4190. 10.1021/acs.jcim.9b01162. 32573225

[ref31] Boldini D.; Friedrich L.; Kuhn D.; Sieber S. A. Tuning Gradient Boosting for Imbalanced Bioassay Modelling with Custom Loss Functions. J. Cheminformatics 2022, 14 (1), 80 10.1186/s13321-022-00657-w. PMC9650867 36357942

[ref32] Jiang D.; Wu Z.; Hsieh C.-Y.; Chen G.; Liao B.; Wang Z.; Shen C.; Cao D.; Wu J.; Hou T. Could Graph Neural Networks Learn Better Molecular Representation for Drug Discovery? A Comparison Study of Descriptor-Based and Graph-Based Models. J. Cheminformatics 2021, 13 (1), 12 10.1186/s13321-020-00479-8. PMC7888189 33597034

[ref33] Zhao W.; Hevener K. E.; White S. W.; Lee R. E.; Boyett J. M. A Statistical Framework to Evaluate Virtual Screening. BMC Bioinformatics 2009, 10 (1), 225 10.1186/1471-2105-10-225. 19619306 PMC2722655

[ref34] Butkiewicz M.; Wang Y.; Bryant S. H.; Lowe E. W. Jr.; Weaver D. C.; Meiler J. High-Throughput Screening Assay Datasets from the PubChem Database. Chem. Inf. (Wilmington, Del) 2017, 3 (1), 1 10.21767/2470-6973.100022. PMC5962024 29795804

[ref35] Buterez D.; Janet J. P.; Kiddle S. J.; Liò P. MF-PCBA: Multifidelity High-Throughput Screening Benchmarks for Drug Discovery and Machine Learning. J. Chem. Inf. Model. 2023, 63 (9), 2667–2678. 10.1021/acs.jcim.2c01569. 37058588 PMC10170507

[ref36] Chakravorty S. J.; Chan J.; Greenwood M. N.; Popa-Burke I.; Remlinger K. S.; Pickett S. D.; Green D. V. S.; Fillmore M. C.; Dean T. W.; Luengo J. I.; Macarrón R. Nuisance Compounds, PAINS Filters, and Dark Chemical Matter in the GSK HTS Collection. SLAS Discovery 2018, 23 (6), 532–544. 10.1177/2472555218768497. 29699447

[ref37] Walters W. P.; Namchuk M. Designing Screens: How to Make Your Hits a Hit. Nat. Rev. Drug Discovery 2003, 2 (4), 259–266. 10.1038/nrd1063. 12669025

[ref38] Sharchilev B.; Ustinovsky Y.; Serdyukov P.; de Rijke M. Finding Influential Training Samples for Gradient Boosted Decision Trees. arXiv, March 12, 2018. http://arxiv.org/abs/1802.06640 (accessed 2022-07-29).

[ref39] Liu F. T.; Ting K. M.; Zhou Z.-H. Isolation Forest. In 2008 Eighth IEEE International Conference on Data Mining; IEEE: Pisa, Italy, 2008; pp 413–422. 10.1109/ICDM.2008.17.

[ref40] Pedregosa F. Scikit-Learn: Machine Learning in Python. J. Machine Learning Res. 2011 12 2825

[ref41] Albuquerque Filho J. E. D.; Brandao L. C. P.; Fernandes B. J. T.; Maciel A. M. A. A Review of Neural Networks for Anomaly Detection. IEEE Access 2022, 10, 112342–112367. 10.1109/ACCESS.2022.3216007.

[ref42] Kingma D. P.; Welling M. Auto-Encoding Variational Bayes. arXiv December 10, 2022. 10.48550/arXiv.1312.6114.

[ref43] Ruff L.; Kauffmann J. R.; Vandermeulen R. A.; Montavon G.; Samek W.; Kloft M.; Dietterich T. G.; Muller K.-R. A Unifying Review of Deep and Shallow Anomaly Detection. Proc. IEEE 2021, 109 (5), 756–795. 10.1109/JPROC.2021.3052449.

[ref44] Gómez-Bombarelli R.; Wei J. N.; Duvenaud D.; Hernández-Lobato J. M.; Sánchez-Lengeling B.; Sheberla D.; Aguilera-Iparraguirre J.; Hirzel T. D.; Adams R. P.; Aspuru-Guzik A. Automatic Chemical Design Using a Data-Driven Continuous Representation of Molecules. ACS Cent. Sci. 2018, 4 (2), 268–276. 10.1021/acscentsci.7b00572. 29532027 PMC5833007

[ref45] Stork C.; Mathai N.; Kirchmair J. Computational Prediction of Frequent Hitters in Target-Based and Cell-Based Assays. Artif. Intell. Life Sci. 2021, 1, 100007 10.1016/j.ailsci.2021.100007.

[ref46] Alves V. M.; Capuzzi S. J.; Braga R. C.; Korn D.; Hochuli J. E.; Bowler K. H.; Yasgar A.; Rai G.; Simeonov A.; Muratov E. N.; Zakharov A. V.; Tropsha A. SCAM Detective: Accurate Predictor of Small, Colloidally Aggregating Molecules. J. Chem. Inf. Model. 2020, 60 (8), 4056–4063. 10.1021/acs.jcim.0c00415. 32678597

[ref47] Senger M. R.; Fraga C. A. M.; Dantas R. F.; Silva F. P. Filtering Promiscuous Compounds in Early Drug Discovery: Is It a Good Idea?. Drug Discovery Today 2016, 21 (6), 868–872. 10.1016/j.drudis.2016.02.004. 26880580

[ref48] Dantas R. F.; Evangelista T. C. S.; Neves B. J.; Senger M. R.; Andrade C. H.; Ferreira S. B.; Silva-Junior F. P. Dealing with Frequent Hitters in Drug Discovery: A Multidisciplinary View on the Issue of Filtering Compounds on Biological Screenings. Expert Opin. Drug Discovery 2019, 14 (12), 1269–1282. 10.1080/17460441.2019.1654453. 31416369

[ref49] Bollinger S. R.; Engers D. W.; Ennis E. A.; Wright J.; Locuson C. W.; Lindsley C. W.; Blakely R. D.; Hopkins C. R. Synthesis and Structure-Activity Relationships of a Series of 4-Methoxy-3-(Piperidin-4-Yl)Oxy Benzamides as Novel Inhibitors of the Presynaptic Choline Transporter. Bioorg. Med. Chem. Lett. 2015, 25 (8), 1757–1760. 10.1016/j.bmcl.2015.02.058. 25801932 PMC4385452

[ref50] Wassermann A. M.; Lounkine E.; Hoepfner D.; Le Goff G.; King F. J; Studer C.; Peltier J. M; Grippo M. L; Prindle V.; Tao J.; Schuffenhauer A.; Wallace I. M; Chen S.; Krastel P.; Cobos-Correa A.; Parker C. N; Davies J. W; Glick M. Dark chemical matter as a promising starting point for drug lead discovery. Nat. Chem. Biol. 2015, 11, 958–966. 10.1038/nchembio.1936. 26479441

