# Supplementary material for: Machine Learning Assisted Hit Prioritization for High Throughput Screening in Drug Discovery
Source: ACS Cent Sci. 2024 Mar 15;10(4):823–32. doi: 10.1021/acscentsci.3c01517 (PMC11046457; doi:10.1021/acscentsci.3c01517)
Supplement: Supplementary file 1 — oc3c01517_si_001.pdf [file oc3c01517_si_001.pdf]

Supporting Information

Machine learning assisted hit prioritization for high throughput screening in drug discovery

Davide Boldini,[†] *Lukas Friedrich,*[§] *Daniel Kuhn*[§] *and Stephan A. Sieber*[*,†]

† Technical University of Munich, TUM School of Natural Sciences, Department of Bioscience, Center for Functional Protein Assemblies (CPA), 85748 Garching bei München, Germany

§ The Healthcare business of Merck KGaA, Darmstadt, Germany

*Email: stephan.sieber@tum.de

1. Minimum Variance Sampling Analysis (MVS-A)

Typically, the identification of mislabeled samples is performed via anomaly detection algorithms. These approaches are usually used in an unsupervised or semi-supervised fashion, where e.g. given a class of compounds and biological readouts, the goal is to identify molecules with noisy measurements.[1,2] In the case of HTS, one example application could be to fit the

anomaly detection algorithm on the hits of the screen and then flag compounds that are particularly dissimilar compared to the distribution of active compounds.

Data valuation methods instead first fit a supervised classification model on the HTS data to distinguish active compounds from inactives. Then, these algorithms estimate which active compounds are particularly problematic for the supervised classification model to reconcile with the rest of the class, thus flagging them as potential false positives.[3] As such, the key difference is the use of a supervised classifier beforehand, thus providing the binary class boundary as an inductive bias for cleaning the training data.

In this context, monitoring sample gradients during training has proved to be an effective data valuation approach to clean noisy datasets.[3–8] For example, TracIn,[3] a recently developed data valuation approach, has demonstrated that tracing sample loss gradients at different checkpoints while training a neural network enables identification of incorrect labels, even without a clean validation set. Monitoring sample gradients works for this application because corrupted data is likely to have large gradients, while correctly labeled instances will have small gradients.[3] As the former require memorization to be learned during training, they strongly affect the neural network weights, while the latter can be easily identified by exploiting the patterns inferred from other clean samples. However, one key limitation of TracIn is that computing all partial derivatives can be prohibitively expensive and approximating this process by focusing on specific network layers (i.e. the last one) can cause performance issues.[5] Furthermore, training and optimization of neural networks can be especially challenging on large, imbalanced molecular datasets such as HTS data,[9,10] while their sensitivity to hyperparameters and architecture make it difficult to provide robust out-of-the-box configurations.

To tackle these limitations we have developed Minimum Variance Sampling Analysis (MVS-A), a data valuation approach based on gradient boosting. Relying on gradient boosting as the underlying algorithm provides the following advantages:

1. Faster training and inference of mislabeling scores.
2. Robust hyperparameter configuration.[11,12]
3. Excellent classification performance on imbalanced HTS data, thus providing a good inductive bias for detecting mislabeled samples.[9,13]

Similar to TracIn, our method first trains a predictor on the noisy dataset, and then flags samples that disproportionately influence the model's parameters as mislabeled. However, except for the decision tree leaf weights, gradient boosting does not have trainable weights. As such, using the original formulation of TracIn in gradient boosting would only evaluate sample influence on the leaf weights, but not on the decision tree structure. Furthermore, there are already heuristics available to compute the impact of a given training instance on the leaf weights of a gradient boosting model to detect mislabeled samples.[14] Instead, we hypothesize that monitoring sample influence on the decision tree structure provides a more global estimation of importance and might provide a better inductive bias for anomaly retrieval. This would be consistent with Isolation Forest, a well established unsupervised OOD detection algorithm based on decision trees.[13]

As such, we replace the original definition of influence from TracIn with the Minimal Variance Sampling (MVS) probability.[16] This estimate was originally developed in the context of stochastic gradient boosting for sampling data instances so that the resulting tree splits would be as similar as possible with the ones obtained by training on the whole dataset. Therefore, samples with high MVS probabilities are the ones contributing the most to a given decision tree structure,

making it an appropriate measure of training data influence. Putting it all together, MVS-A scores can be computed as follows:

$$MVS\text{-}A_j = \sum_{i}^{n} min\left(1, \sqrt{{g_{i_j}}^2 + \lambda {h_{i_j}}^2}\right)$$

Where n is the number of trees in the ensemble, g_i and h_i are the loss function gradient and hessian at boosting iteration i for sample j and λ is the L1 norm of the leaf weights of the previous tree. In other words, MVS-A adopts the same theoretical framework of TracIn with the following modifications:

1. Sample influence on the training process is quantified as MVS probability.
2. Each boosted tree is used as a checkpoint to monitor the influence of a sample across the training process.

2. CatBoost object importance

Sharchilev and coworkers have shown that it is possible to obtain valid sample importance scores for a given gradient boosting model by evaluating the influence a given sample has on the leaf weights in the ensemble.[14] This method is much faster than Leave-One-Out retraining since it involves perturbing one leaf weight per tree in the ensemble, under the assumption that the tree splits would stay constant if that sample were to be excluded from training. In practice however, computing sample influence with this method requires a clean validation set: the idea is that correctly labeled samples reduce the loss when predicting clean samples, while mislabeled samples in the training set decrease performance. Since our benchmark did not include the presence of a clean validation set, we used all actives in the training set as the

external dataset to use for tracking loss changes. Computing object importance in this way makes it conceptually similar to TracIn self-proponent calculation, where false positives strongly contribute in decreasing their own loss, thus leading to memorization, due to their inconsistency with other bioactive compounds.

3. Dataset collection

We report for each HTS dataset the readouts and activity cutoffs in Table S1, the biological information of the targets in Table S2 and the PubChem AIDs in Table S3. The complete table and additional dataset statistics (i.e. number of actives, inactives, false positives, true positives and so forth) are available at https://github.com/dahvida/AIC_Finder.

Table S1 – Measurement details for each publicly available HTS used in this study.

Name	Primary readout	Confirmatory measurement	Confirmatory cutoff
transporter	Inhibition at 6.6 uM	2 Replicates	46.00%
transcription	Inhibition at 3.6 uM	2 Replicates	20.00%
transcription_2	B score at 10 uM	2 Replicates	3SD from blank
GPCR_2	B score at 10 uM	2 Replicates	3SD from blank
GPCR_3	B score at 10 uM	EC50	Failed fit
ion_channel	B score at 10 uM	2 Replicates	3SD from blank
ion_channel_2	Inhibition at 10 uM	EC50	100 uM or failed fit
ion_channel_3	B score at 10 uM	2 Replicates	3SD from blank
kinase	Activity at 12.5 uM	AC50	350 uM or failed fit
GPCR	Activity at 2.5 uM	EC50	195 uM or failed fit
serine	Inhibition at 21.8 uM	IC50	Equation (pIC50, QC)
transcription_3	Inhibition at 20 uM	IC50	Equation (pIC50, QC)
ubiquitin	Inhibition at 5 uM	IC50	50 uM or failed fit
splicing	Activity at 7.5 nM	EC50	380 uM or failed fit

channel_atp	Activity at 7.5 uM	AC50	100 uM or failed fit
cysteine_protease	Activity at 12.5 uM	AC50	70 uM or failed fit
zinc_finger	Activity at 10 uM	EC50	300 uM or failed fit

Table S2 – biological target information for each publicly available HTS used in this study.

Name	Target Uniprot ID	Protein family
transporter	O43613	Transporter
transcription	P25929	Transcription
transcription_2	P08482	Transcription
GPCR_2	P35561	GPCR
GPCR_3	O95180	GPCR
ion_channel	O88943	Ion channel
ion_channel_2	Q9BYT3	Ion channel
ion_channel_3	Q9GZV3	Ion channel
kinase	CAD53472	Kinase
GPCR	AAH94064	GPCR
serine	P34707	Protease
transcription_3	P61088	Transcription
ubiquitin	Q9Y337	Ubiquitin
splicing	P9WHJ3	Splicing
channel_atp	P13569	Ion channel
cysteine_protease	P55212	Protease
zinc_finger	Q9XUB2	Zinc finger

Table S3 – PubChem assay identifiers for each publicly available HTS investigated in this study.

Name	Primary AID	Confirmatory AID
transporter	485270	492964
transcription	1040	1254

transcription_2	628	677
GPCR_2	1672	2032
GPCR_3	449739	489005
ion_channel	2239	2287
ion_channel_2	2661	2821
ion_channel_3	488975	493221
kinase	504558	588343
GPCR	2098	2382
serine	624304	624474
transcription_3	485273	493155
ubiquitin	873	1431
splicing	2221	435010
channel_atp	720511	743267
cysteine_protease	686996	720632
zinc_finger	1832	1960

Table S4 – Scaffold diversity percentage, expressed as the ratio between the number of unique Murcko scaffolds and the total number of compounds, of hits and non-hits for each publicly available HTS investigated in this study.

Name	Scaffold diversity % - actives	Scaffold diversity % - inactives
GPCR	66.5	28.1
GPCR_2	79.2	34.9
GPCR_3	84.3	49.5
channel_atp	82.7	22.6
cysteine_protease	75.9	23.4
ion_channel	62.0	28.5
ion_channel_2	74.5	41.7
ion_channel_3	67.1	28.4

kinase	74.8	28.0
serine	86.9	34.0
splicing	58.3	29.0
transcription	77.8	27.5
transcription_2	83.4	28.6
transcription_3	79.8	27.6
transporter	77.1	28.5
ubiquitin	61.8	27.5
zinc_finger	76.5	28.5

4. Chemical space analysis

We qualitatively assessed the diversity of the primary HTS hits present in our collection via supervised dimensionality reduction with Uniform Manifold Approximation and Projection (UMAP),[17] using Extended Connectivity Fingerprints (ECFP)[18] as molecular representation. For some protein target families, it is possible to cluster the hits from the primary assays, for example in the case of GPCR and transcription factors (Figure S1a). In other cases, the active compounds tend to be remarkably different from each other (e.g. for the Kinase dataset).

Overall, active compounds within the same dataset have an average Tanimoto similarity of 0.135 ± 0.006, while when comparing different datasets they have a value of 0.128 ± 0.005, highlighting the high chemical diversity present in our HTS selection. The low average Tanimoto similarity, both within and between datasets, is also consistent with the lack of visible clusters when using UMAP in an unsupervised fashion (Figure S1b).

a)

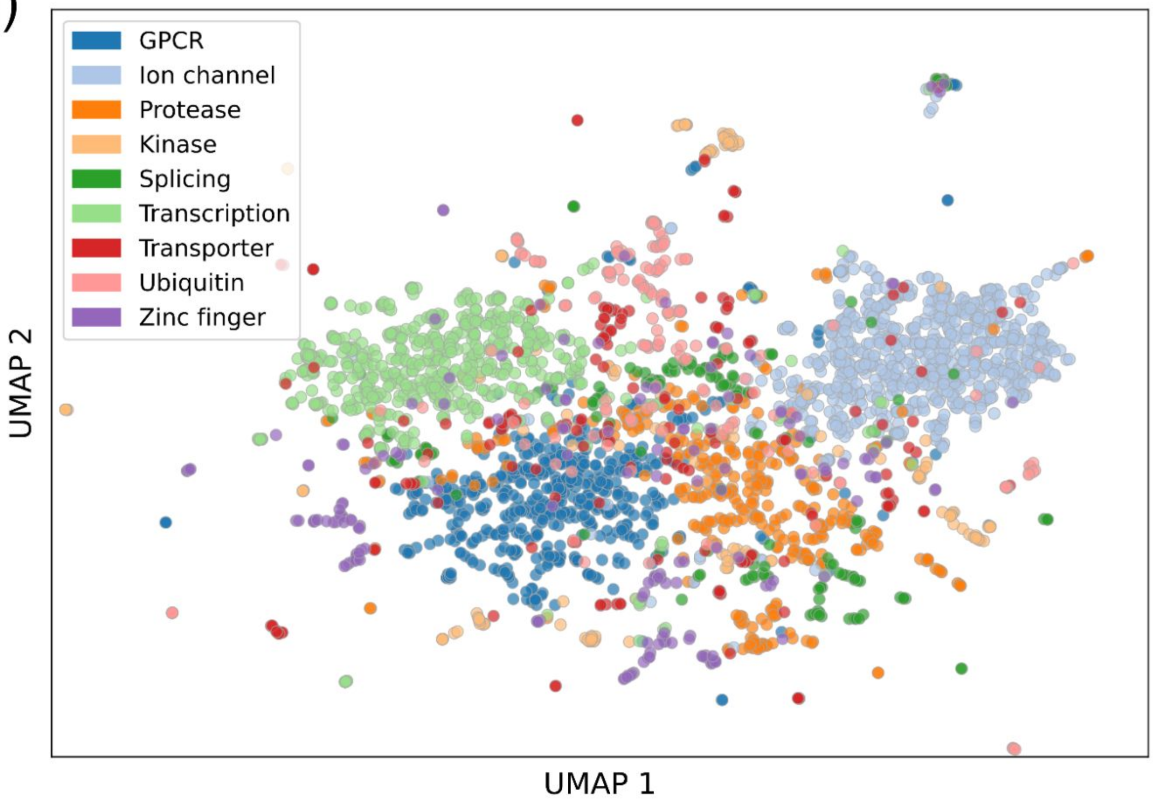

b)

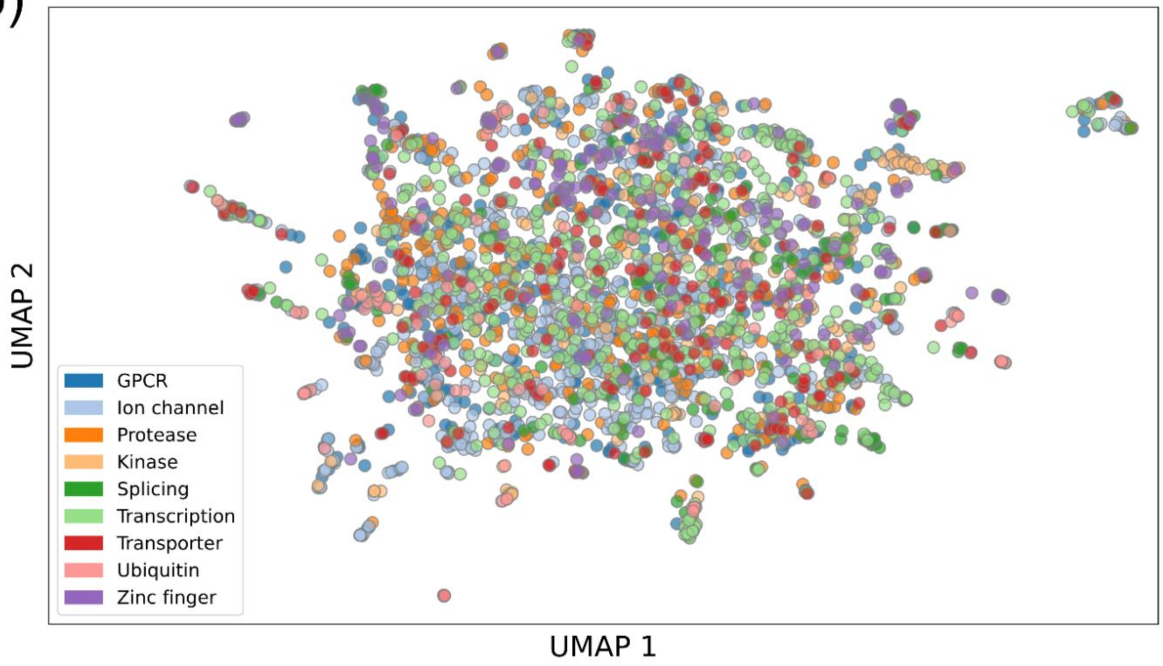

Figure S1 – Chemical space covered by the hits of the high throughput screening datasets used in this study. a) ECFP dimensionality reduction using supervised UMAP. b) ECFP dimensionality reduction using unsupervised UMAP.

5. Methods

5.1. Performance metrics

- **Top-K precision**: Indicates the fraction of compounds in the top-K percentile of the ranking that are true positives or true negatives. When compared to the true positive or false positive rate of an assay, it measures the improvement a given prioritization strategy provides over randomly selecting compounds. In this study, K was set to the top 10% of the ranked list.
- **Top-K enrichment factor**: it is a variant of top-K precision, but it is normalized against the total number of true positives or true negatives and the assay size.[19] It is calculated as follows:

$$EF = \frac{H_k}{N_k} * \frac{H_t}{N_t}$$

  Where H_k is the number of hits in the top-K of the ranking, N_k is the number of compounds in the top-K percentile, H_t is the total number of hits and N_t is the total number of compounds in the HTS dataset. In this study, K was set to the top 10% of the ranked list.
- **BEDROC**: it is a variation of the ROC-AUC score, where the value can be more or less dependent on how accurate the prediction is for the top-K samples depending on the α hyperparameter.[19] In this study, we used the default configuration suggested in the original paper of $\alpha = 20$. With this parameter, 80% of the score depends on the detection performance of the top 20% of the list, while the rest of the ranking influences the remaining 20% of the score. As such, this metric is complementary to precision and enrichment factor since it focuses on a different top-K percentile while still measuring the performance at lower priority scores.

5.2. Model construction and evaluation

For MVS-A, we chose LightGBM[20] to construct the gradient boosting classification models, using 100 boosting iterations, 1.0 lambda regularization and 0.95 column subsampling for all datasets. Lambda regularization provided marginal improvements in false positive and true positive detection, which we assume is due to its influence on the hessian weight when calculating MVS probabilities. Column subsampling was necessary to add randomness to the training process for metric uncertainty estimation. We used Extended Connectivity Fingerprints (ECFP) with 1024 bits and radius 2 for encoding molecular structures in a numerical format.[18] After training the classifier on the primary assay labels, we computed the MVS-A scores for each compound according to the formula in Section 1. In order to compute normalized precision and enrichment factor, we selected the top 10% and bottom 10% of the scores for false positive and true positive detection respectively. However, given the high level of class imbalance in the datasets, minority class gradients tend to be much larger than the ones for the majority class, thus leading to two different MVS-A distributions for active and inactive compounds. Because of this, percentile calculations were done class-wise. Once top-K predictions and raw MVS-A scores were calculated for all primary compounds, the performance metrics were computed for the subset of compounds that were considered active in the primary screen and were further tested in the confirmatory assay. Since the top-K prediction thresholds are calculated with only primary screening information available, this ensures that the analysis is not biased by knowing which compounds were selected for the confirmatory screen.

Regarding CatBoost object importance, we followed the same overall benchmark procedure as for MVS-A. First, we trained a CatBoost[21] classifier on the primary screen data with the default hyperparameters of the GBM package, using ECFPs with 1024 bits and radius 2. Then, since

for this type of benchmark there is no clean validation set available, we selected all primary actives as the validation set to use for influence calculation. We used the default hyperparameters of the “get_object_importance” method from the CatBoost package to compute the scores. Once the CatBoost object importance scores are calculated, the rest of the analysis is performed in the same way as for MVS-A.

For primary readout ranking, we sorted all compounds that were active in the primary screen according to the reported “PubChem activity score”. Then, we selected the top 10% as top-K predictions for true positive detection. Next, we selected the subset of compounds that were also evaluated in the confirmatory screen and we computed the performance metrics.

We implemented GSK and REOS structural alerts using RDKIT.[22] Compounds are ranked according to the number of matches they have with either collection of filters, with more flags indicating a higher likelihood of being a false positive. Once all primary actives were processed in this way, we measured performance metrics on the subset of compounds that were tested again in the confirmatory screen.

Concerning Isolation Forest, given a HTS dataset, we fit the model on all primary hits using ECFPs with 1024 bits and radius 2, then computed the anomaly scores for each sample in the training set. Compounds with high anomaly scores were deemed false positives and vice-versa for true positives. Once all primary actives were processed in this way, we measured performance metrics on the subset of compounds that were tested again in the confirmatory screen. Each model was fit using scikit-learn’s default parameters.

Finally, we developed VAE anomaly detection models using the same procedure used for Isolation Forest. Compounds with high reconstruction error were deemed false positives and vice-versa for true positives. Once all primary actives were processed in this way, we measured

performance metrics on the subset of compounds that were tested again in the confirmatory screen. For each model, we used SMILES one-hot encodings as input and the architecture described by Gómez-Bombarelli et al.

For the raw results for each method in each dataset, please refer to https://github.com/dahvida/AIC_Finder.

5.3. Auto-fluorescence predictor development

We downloaded the training dataset from Borrel et al. Since the authors only reported the CAS identifiers for each compound, we used the Chemical Identifier Resolver to convert CAS identifiers to SMILES. Only approximately half of the compounds (4065) were possible to parse in this way. We selected all cell-based and cell-free fluorescence assays, for a total of 12 classification tasks. As described in the original publication, each task is split into 90:10 train-test splits using stratified random splitting. However, since the authors do not report the splits they used, we cannot exactly reproduce their test sets.

We featurized each compound using Mordred, computing all 1D and 2D molecular descriptors, for a total of 1613 features per molecule. This selection encompasses all molecular descriptors used for InterPred. Unfortunately, we could not use the same package used by the authors since it requires Python 2, while our study requires Python 3.

We modeled each dataset using Random Forest as described in the original publication, using scikit-learn's implementation. For each task, we carry out hyperparameter optimization via grid search on 10-fold cross validation on the training test. Since there is no information available on the optimization routine, we chose to optimize the cross-validation PR-AUC, tuning the number of estimators and maximum number of features.

To tackle class imbalance, the final model for each task consists of an ensemble of 10 Random Forest classifiers trained on resampled versions of the training set, as described in the original publication. Each version is generated via random undersampling, enforcing a 70:30 ratio between inactives and actives for a given task.

For the false positive detection benchmark, compounds are sorted in terms of auto-fluorescence predicted probability.

The performance achieved by the auto-fluorescence predictor for each task is available at https://github.com/dahvida/AIC_Finder.

5.4. Hit Dexter and SCAM Detective

We used batch processing via the Hit Dexter webserver (https://nerdd.univie.ac.at/hitdexter3/) to compute frequent hitter predictions for each dataset evaluated in this study. For the false positive detection benchmark, we sorted compounds in terms of likelihood of being a highly promiscuous compound (column "HPROM-NPROM target-based assay data set model" from the csv output).

We downloaded the SCAM Detective models from the respective Github repository (https://github.com/alvesvm/scam_detective), since the webapp does not support batch processing. For the false positive detection benchmark, we sorted compounds in terms of global likelihood of being a colloidal aggregator, obtained by averaging the predictions of the Cruzain and β-Lactamase models.

6. False positive and true positive detection benchmark

Table S5 – Bonferroni corrected p-values of the one-tailed Wilcoxon Signed Rank tests between MVS-A and a given baseline across all datasets for false positive detection.

Baseline	Relative Precision	Enrichment Factor	BEDROC
CatBoost	0.000267029	0.000534058	0.003738403
Structural filters	5.34E-05	5.34E-05	0.00037384
Isolation Forest	0.000160217	0.000160217	0.000106812
VAE	0.000267029	0.000160217	0.000267029
Auto-fluorescence	5.34E-04	5.34E-04	7.48E-04
Hit Dexter	5.34E-05	5.34E-05	5.34E-05
SCAM Detective	2.67E-04	5.34E-04	1.60E-04

Table S6 – Bonferroni corrected p-values of the one-tailed Wilcoxon Signed Rank tests between MVS-A and a given baseline across all datasets for false positive detection.

Baseline	Relative Precision	Enrichment Factor	BEDROC
CatBoost	0.000579834	0.00100708	3.05E-05
Primary score	0.009277344	0.000762939	0.015808105
Isolation Forest	6.10E-05	6.10E-05	6.10E-05
VAE	0.000427246	0.000427246	0.000152588

7. Overlap calculation for MVS-A predictions

We computed the overlap between MVS-A and other in silico tools by first selecting all compounds predicted to be false positives by our tool, then calculating the fraction of those that were also identified by another method. For GSK and REOS filters, we considered a compound to be an overlap if it was flagged by at least one alert. For the remaining models, we counted compounds as overlaps if the alternative method predicted them to be interferents with a probability score higher than 0.5.

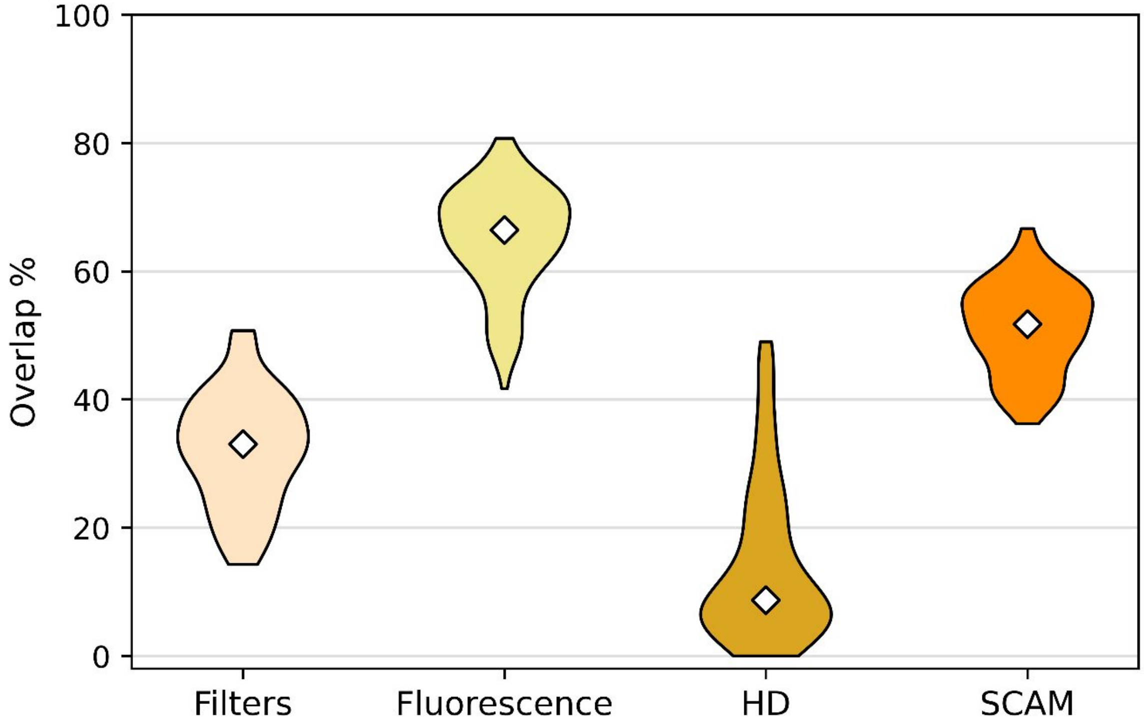

Figure S2 – Fraction of false positives detected both by MVS-A and a given alternative method. The distribution of the overlap score is evaluated across all HTS datasets.

Table S7 – Percentage of primary actives flagged by each in-silico false positive predictor across each dataset evaluated in this study.

Name	Auto-fluorescence	SCAM Detective	Hit Dexter	Filters
GPCR	86.1	55.6	13.7	45.2
GPCR 2	72.0	66.2	2.5	40.3
GPCR 3	59.2	61.0	2.0	23.0
channel atp	82.4	66.3	11.7	47.4
cysteine protease	77.3	47.6	32.0	39.2
ion channel	57.3	53.6	9.5	47.9
ion channel 2	80.0	66.6	2.9	35.4
ion channel 3	65.3	48.7	10.9	50.1
kinase	90.2	56.3	35.7	65.6
serine	66.7	48.7	4.6	34.4
splicing	81.4	51.5	30.9	59.3
transcription	75.4	60.1	9.8	47.4
transcription 2	69.2	51.8	6.1	39.7
transcription 3	78.6	59.9	6.4	34.1
transporter	71.8	54.8	7.0	43.4
ubiquitin	85.0	72.1	10.9	63.0
zinc finger	86.1	61.8	37.5	67.7

8. Merck KGaA datasets

Table S8 – Dataset information for the industry datasets from Merck KGaA. Due to confidentiality issues, only limited information regarding these screens can be disclosed.

Name	Primary screen size	Confirmatory measurement	False positive rate	Number of hits	Target
HTS_1	>350.000	IC50	N.A.	N.A.	N.A.
HTS_2	>350.000	IC50	N.A.	N.A.	N.A.
HTS_3	>350.000	IC50	N.A.	N.A.	N.A.

Table S9 – Mean false positive detection performance for all algorithms on the Merck KGaA datasets. Each measurement is reported as a mean of 10 replicates. If the difference between MVS-A and all other baselines is statistically significant according to a one-tailed Wilcoxon Signed Rank test (α=0.05) with Bonferroni correction, the value is highlighted in bold.

Algorithm	Relative precision *(k=10)*	Enrichment factor *(k=10)*	BEDROC *(α=20)*
MVS-A	**0.17 ± 0.01**	0.25 ± 0.01	**0.57 ± 0.01**
CatBoost	0.06 ± 0.03	0.24 ± 0.01	0.47 ± 0.04

Table S10 – True positive detection performance for all algorithms on the Merck KGaA datasets. Each measurement is reported as a mean of 10 replicates. If the difference between MVS-A and all other baselines is statistically significant according to a one-tailed Wilcoxon Signed Rank test (α=0.05) with Bonferroni correction, the value is highlighted in bold.

Algorithm	Relative precision *(k=10)*	Enrichment factor *(k=10)*	BEDROC *(α=20)*
MVS-A	0.14 ± 0.01	0.50 ± 0.01	0.74 ± 0.01
CatBoost	0.14 ± 0.02	0.49 ± 0.01	0.75 ± 0.02
Primary readout	0.17	0.50	0.77

9. Practical guidelines for the use of MVS-A

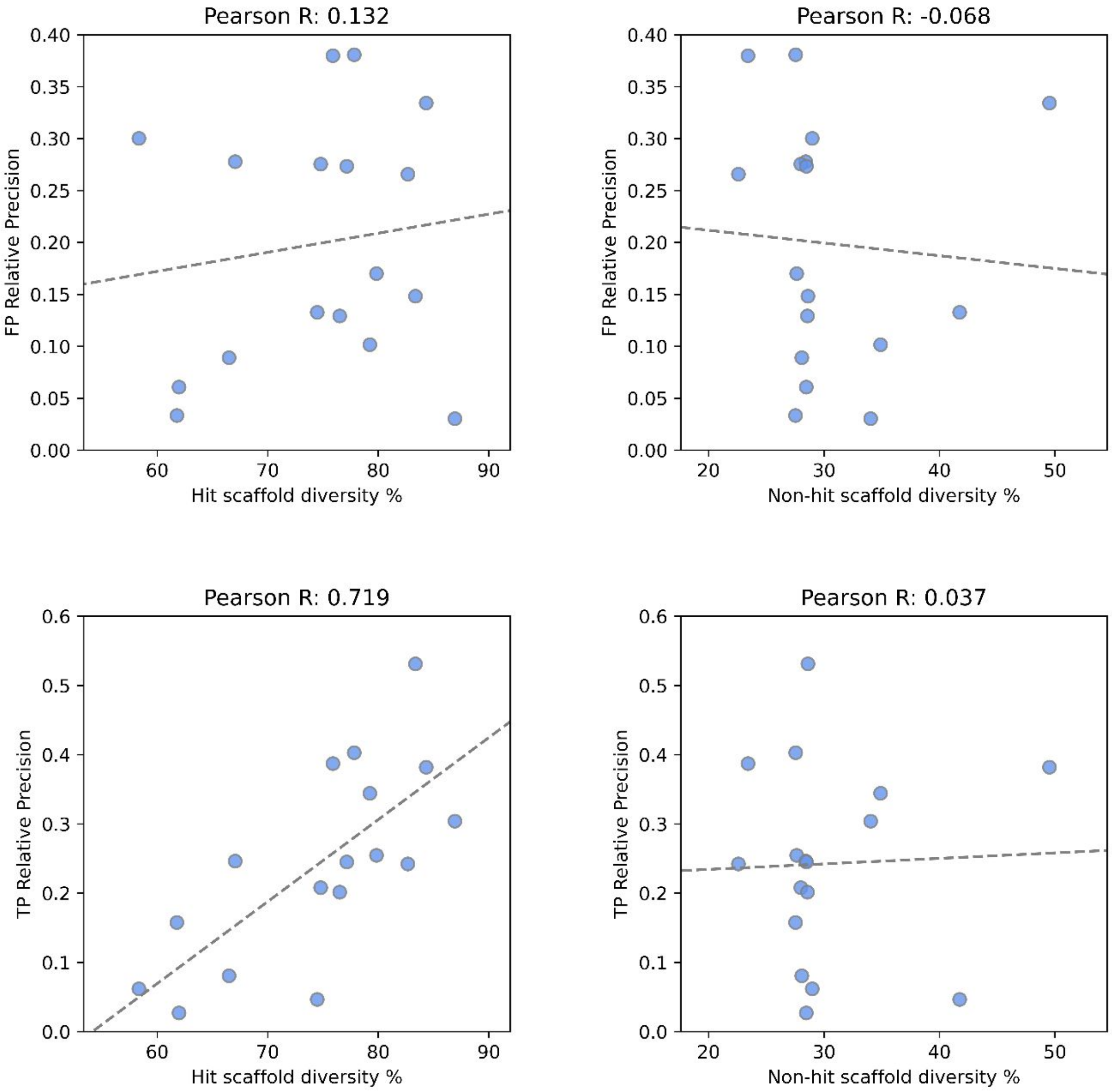

Figure S3 – Correlation analysis between true positive relative precision, false positive relative precision, scaffold diversity of active compounds and scaffold diversity of inactive compounds.

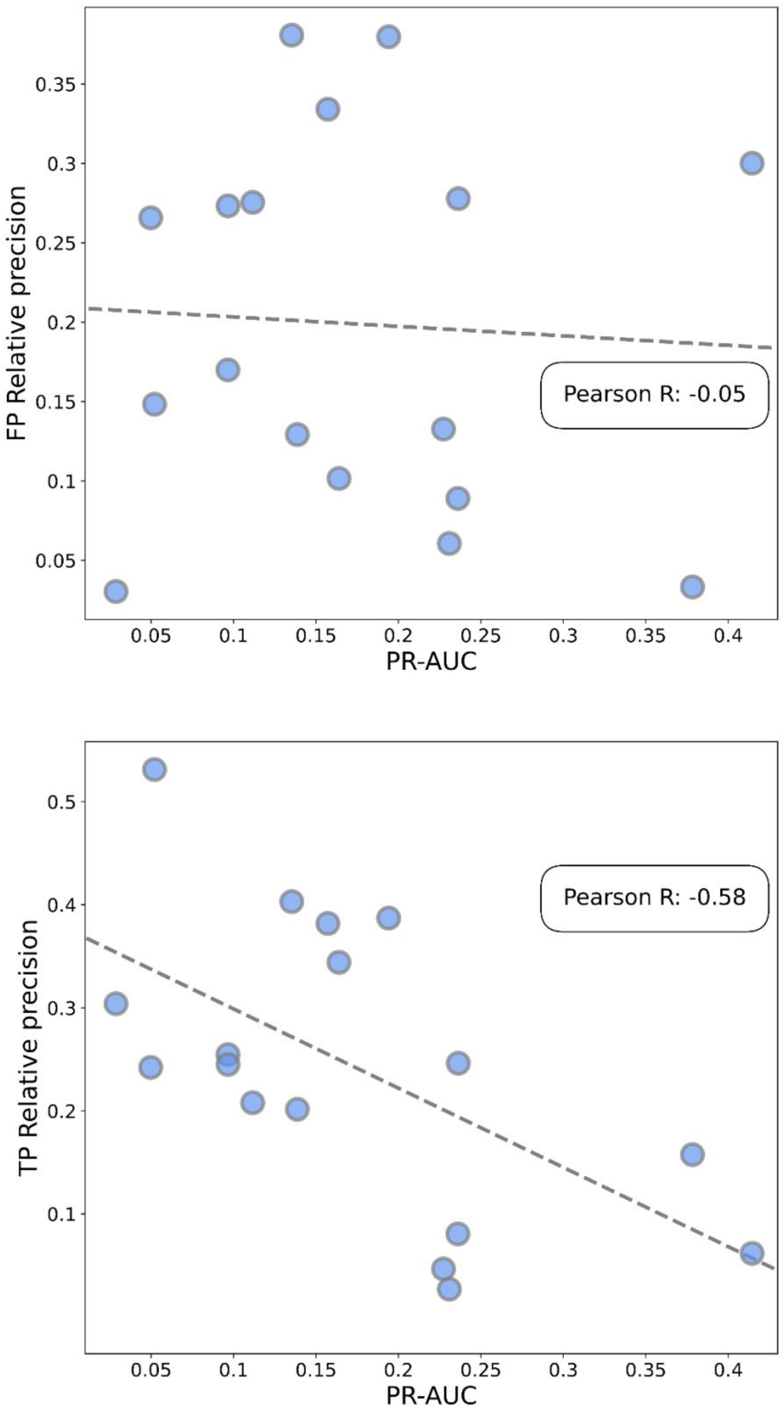

Figure S4 – Correlation analysis between classification performance (expressed as PR-AUC in 5-fold cross validation) and true positive and false positive detection performance (expressed as relative precision) for each dataset investigated in this study.

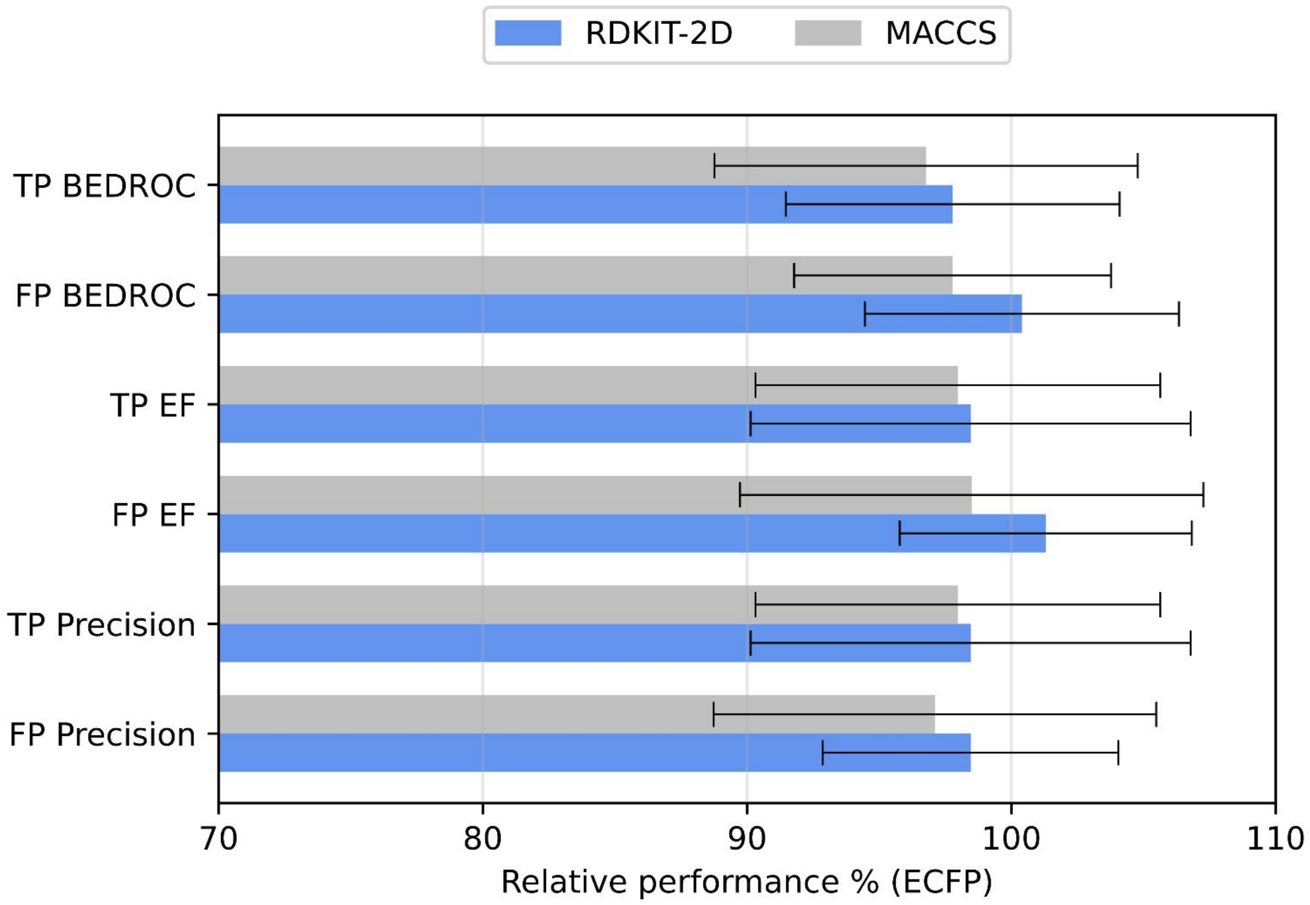

Figure S5 - Mean relative MVS-A performance of alternative molecular representations across all datasets. Standard deviation refers to the inter-dataset variability. We replicated the analysis procedure used for MVS-A using either MACCS keys or 207 2D molecular descriptors as input representations. Both alternative representations were generated using RDKIT.

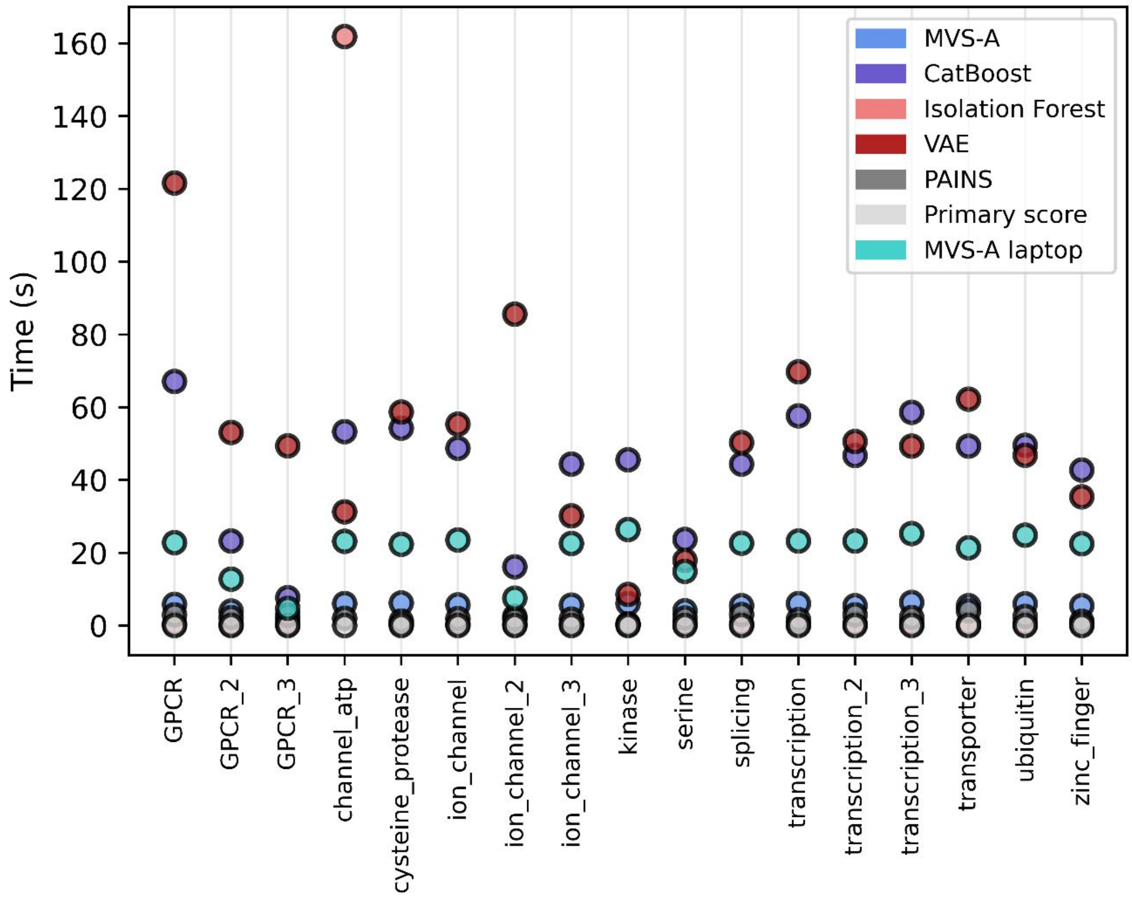

Figure S6 – Training time required by each method evaluated in this benchmark. All calculations were carried out on an AMD Ryzen Threadripper 3970 CPU with 32 cores, with the exception of MVS-A laptop, which were performed on an AMD Ryzen 5 3600 6-Core Processor.

Table S10 – Reanalysis of the top 20 ranked hits for Case Study I according to MVS-A. The "True hit" column denotes whether a given compound was a true hit from the screening campaign.

SMILES	MVS-A	True hit	Filter alerts	Hit Dexter	SCAM Detective	InterPred
COc1ccc(C(=O)NCCCSC)cc1OC1CCN(C(C)C)CC1	41.2	0	1	0	1	Error
COc1ccc(C(=O)NC(Cn2ccnc2)C(C)(C)C)cc1OC1CCN(C(C)C)CC1	45.4	0	0	0	1	Error
COc1ccc(C(=O)NCc2cnn(C)c2C)cc1OC1CCN(C(C)C)CC1	50.7	1	0	0	1	Error
COc1ccc(C(=O)NCc2nc(C)c(C)s2)cc1OC1CCN(C(C)C)CC1	50.9	0	0	0	1	Error
COc1ccc(C(=O)NCCC2=CCCCC2)cc1OC1CCN(C(C)C)CC1	51.0	0	0	0	1	Error
COc1ccc(C(=O)NC2CCCCC2)cc1OC1CCN(C(C)C)CC1	51.7	0	0	0	0	Error
O=C(Nc1ccc(S(=O)(=O)Nc2cnc3ccccc3n2)cc1)c1ccc(Cl)cc1Cl	52.1	0	0	0	0	1
COc1cccc(CCNC(=O)c2ccc(OC)c(OC3CCN(C(C)C)CC3)c2)c1	52.3	0	1	0	1	Error
COc1ccc(C(=O)NC(C)CCn2cccn2)cc1OC1CCN(C(C)C)CC1	52.8	0	1	0	1	Error
COc1ccc(C(=O)NC2CCc3ccccc32)cc1OC1CCN(C(C)C)CC1	53.0	0	0	0	0	Error
COc1ccc(C(=O)NCc2cc(C(C)C)no2)cc1OC1CCN(C(C)C)CC1	53.2	1	0	0	1	Error
COc1ccccc1CCNC(=O)c1ccc(OC)c(OC2CCN(C(C)C)CC2)c1	53.5	0	1	0	1	Error
COc1ccc(C(=O)NC(C)Cc2cnccn2)cc1OC1CCN(C(C)C)CC1	54.7	0	0	0	1	Error
COc1ccc(C(=O)NCc2ccon2)cc1OC1CCN(C(C)C)CC1	55.5	1	0	0	1	Error
COc1ccc(C(=O)NC(C)c2nccs2)cc1OC1CCN(C(C)C)CC1	55.5	1	0	0	1	Error
CCc1cc(CNC(=O)c2ccc(OC)c(OC3CCN(C(C)C)CC3)c2)on1	56.1	0	0	0	1	Error

COc1ccc(C(=O)N[C@H]2CCCCNC2=O)cc1OC1CCN(C(C)C)CC1	56.2	1	0	0	1	Error
CCOCCCNC(=O)c1ccc(OC)c(OC2CCN(C(C)C)CC2)c1	56.6	0	1	0	1	Error
O=C(Nc1ccc(S(=O)(=O)Nc2cnc3ccccc3n2)cc1)c1ccc([N+](=O)[O-])cc1Cl	56.9	0	0	0	0	Error

References

(1) Albuquerque Filho, J. E. D.; Brandao, L. C. P.; Fernandes, B. J. T.; Maciel, A. M. A. A Review of Neural Networks for Anomaly Detection. *IEEE Access* **2022**, *10*, 112342–112367. https://doi.org/10.1109/ACCESS.2022.3216007.

(2) Ruff, L.; Kauffmann, J. R.; Vandermeulen, R. A.; Montavon, G.; Samek, W.; Kloft, M.; Dietterich, T. G.; Muller, K.-R. A Unifying Review of Deep and Shallow Anomaly Detection. *Proc. IEEE* **2021**, *109* (5), 756–795. https://doi.org/10.1109/JPROC.2021.3052449.

(3) Pruthi, G.; Liu, F.; Sundararajan, M.; Kale, S. Estimating Training Data Influence by Tracing Gradient Descent. arXiv November 14, 2020. http://arxiv.org/abs/2002.08484 (accessed 2022-08-31).

(4) Feng, Y.; Tu, Y. Phases of Learning Dynamics in Artificial Neural Networks in the Absence or Presence of Mislabeled Data. *Mach. Learn. Sci. Technol.* **2021**, *2* (4), 043001. https://doi.org/10.1088/2632-2153/abf5b9.

(5) Akyurek, E.; Bolukbasi, T.; Liu, F.; Xiong, B.; Tenney, I.; Andreas, J.; Guu, K. Towards Tracing Knowledge in Language Models Back to the Training Data. In *Findings of the Association for Computational Linguistics: EMNLP 2022*; Association for Computational Linguistics: Abu Dhabi, United Arab Emirates, 2022; pp 2429–2446.

(6) Lu, Y.; Bo, Y.; He, W. Noise Attention Learning: Enhancing Noise Robustness by Gradient Scaling. *Adv. Neural Inf. Process. Syst.* **2022**, *35*, 23164–23177.

(7) Toniato, A.; Schwaller, P.; Cardinale, A.; Geluykens, J.; Laino, T. Unassisted Noise Reduction of Chemical Reaction Datasets. *Nat. Mach. Intell.* **2021**, *3* (6), 485–494. https://doi.org/10.1038/s42256-021-00319-w.

(8) Pleiss, G.; Zhang, T.; Weinberger, K. Q.; Elenberg, E. Identifying Mislabeled Data Using the Area Under the Margin Ranking.

(9) Boldini, D.; Friedrich, L.; Kuhn, D.; Sieber, S. A. Tuning Gradient Boosting for Imbalanced Bioassay Modelling with Custom Loss Functions. *J. Cheminformatics* **2022**, *14* (1), 80. https://doi.org/10.1186/s13321-022-00657-w.

(10) Korkmaz, S. Deep Learning-Based Imbalanced Data Classification for Drug Discovery. *J. Chem. Inf. Model.* **2020**, *60* (9), 4180–4190. https://doi.org/10.1021/acs.jcim.9b01162.

(11) Grinsztajn, L.; Oyallon, E.; Varoquaux, G. Why Do Tree-Based Models Still Outperform Deep Learning on Tabular Data? arXiv July 18, 2022. http://arxiv.org/abs/2207.08815 (accessed 2023-06-20).

(12) Shwartz-Ziv, R.; Armon, A. Tabular Data: Deep Learning Is Not All You Need. *Inf. Fusion* **2022**, *81*, 84–90. https://doi.org/10.1016/j.inffus.2021.11.011.

(13) Jiang, D.; Wu, Z.; Hsieh, C.-Y.; Chen, G.; Liao, B.; Wang, Z.; Shen, C.; Cao, D.; Wu, J.; Hou, T. Could Graph Neural Networks Learn Better Molecular Representation for Drug Discovery? A Comparison Study of Descriptor-Based and Graph-Based Models. *J. Cheminformatics* **2021**, *13* (1), 12. https://doi.org/10.1186/s13321-020-00479-8.
(14) Sharchilev, B.; Ustinovsky, Y.; Serdyukov, P.; de Rijke, M. Finding Influential Training Samples for Gradient Boosted Decision Trees. arXiv March 12, 2018. http://arxiv.org/abs/1802.06640 (accessed 2022-07-29).
(15) Liu, F. T.; Ting, K. M.; Zhou, Z.-H. Isolation Forest. In *2008 Eighth IEEE International Conference on Data Mining*; IEEE: Pisa, Italy, 2008; pp 413–422. https://doi.org/10.1109/ICDM.2008.17.
(16) Ibragimov, B.; Gusev, G. Minimal Variance Sampling in Stochastic Gradient Boosting. arXiv October 29, 2019. https://doi.org/10.48550/arXiv.1910.13204.
(17) McInnes, L.; Healy, J.; Melville, J. UMAP: Uniform Manifold Approximation and Projection for Dimension Reduction. arXiv September 17, 2020. https://doi.org/10.48550/arXiv.1802.03426.
(18) Rogers, D.; Hahn, M. Extended-Connectivity Fingerprints. *J. Chem. Inf. Model.* **2010**, *50* (5), 742–754. https://doi.org/10.1021/ci100050t.
(19) *Evaluating Virtual Screening Methods: Good and Bad Metrics for the "Early Recognition" Problem | Journal of Chemical Information and Modeling*. https://pubs.acs.org/doi/abs/10.1021/ci600426e (accessed 2023-09-06).
(20) Ke, G.; Meng, Q.; Finley, T.; Wang, T.; Chen, W.; Ma, W.; Ye, Q.; Liu, T.-Y. LightGBM: A Highly Efficient Gradient Boosting Decision Tree. In *Advances in Neural Information Processing Systems*; Curran Associates, Inc., 2017; Vol. 30.
(21) Prokhorenkova, L.; Gusev, G.; Vorobev, A.; Dorogush, A. V.; Gulin, A. CatBoost: Unbiased Boosting with Categorical Features. 11.
(22) *RDKit*. https://www.rdkit.org/ (accessed 2021-05-09).
